# Year-round acoustic presence of fin whales southwest of Svalbard suggests mixed-use habitat for feeding and breeding

**DOI:** 10.1038/s41598-025-21785-x

**Published:** 2025-11-28

**Authors:** Angela R. Szesciorka, Patrizia Giordano, Manuel Bensi, Alessandro Nicolai, Aniello Russo, Giacomo Giorli

**Affiliations:** 1 NATO Science and Technology Organization, Centre for Maritime Research and Experimentation, La Spezia, 19126 Italy; 2 National Research Council, Institute of Polar Sciences, Bologna, 40129 Italy; 3 National Institute of Oceanography and Applied Geophysics, Trieste, 34010 Italy

**Keywords:** Marine biology, Marine mammals

## Abstract

Multiple lines of evidence indicate a northward expansion and year-round presence of fin whales especially around Svalbard, an area where highly variable Atlantic Water intrusion is changing the trophic community. Because fin whales are now documented in the high Arctic (≥ 80°N), we hypothesized that this would also increase their presence in lower latitude Arctic regions. We used one year of passive acoustic data collected on a mooring deployed on the continental slope southwest of Svalbard to assess the temporal acoustic presence of fin whales and the relationship with environmental data. Acoustic detections and call diversity were greatest in fall and spring and lowest in summer. The 20 Hz calls were present from July to April and the 130 Hz calls were present from August to April. Both calls peaked in September with smaller, secondary peaks in March. Downsweeps were bimodally present between July and October and between February and June. Linear models indicated more calls when krill and copepod numbers were high and sea ice extent and amphipod numbers were low. A year-round acoustic presence of a potential resident subpopulation of fin whales suggests the region is a mixed-use habitat for foraging and reproductive-related activities.

**Supplementary Information:**

The online version contains supplementary material available at 10.1038/s41598-025-21785-x.

## Introduction

 Our understanding of whale life history, including migration patterns, originated from early whaling records^1–3^. The classic seasonal to-and-from paradigm of migration is often cited for baleen whales who migrate between low latitude winter grounds and high latitude summer grounds^4,5^. However, this is proving to be a more complicated story for most, if not all, baleen whale species. Geijer et al.’s (2016)^6^ review of whale migration identified several baleen whale populations that were migratory, non-migratory, seasonally dispersing, or exhibiting short-range migration. At the same time, the migration and distribution patterns of some baleen whale populations appear to be changing. Changes in timing coincident with increasing ocean temperatures in the Arctic and sub-Arctic regions and decreasing sea ice suggest a response driven by climate change^7–10^. In other cases, what appears to be range expansion may be a return to habitats that whales were once removed from during commercial whaling^11–13^.

Fin whales (*Balaenoptera physalus*) were thought to be seasonal visitors to the high-latitude regions of the North Atlantic^14^, where they feed on krill, copepods, and pelagic fish^15,16^ before moving southwards to temperate regions to breed during winter. However, multiple lines of evidence (e.g., visual survey, telemetry, and acoustic data) indicate a northward expansion. Fin whale visual and acoustic presence has increased in the Barents Sea^17,18^, Greenland Sea and Fram Strait^19,20^, and off western and southern Svalbard^21,22^. Fin whales are now documented as far north as 81.5° N^23,24^ and have shifted to more coastal areas^22^, including the fjords of Svalbard^25^. With some tagged whales remaining in Svalbard weeks after the last known migrating whale had departed^26^ and intermittent acoustic detections throughout the year at higher latitudes^24,27,28^ it is believed that at least a portion of the fin whale population resides in the Svalbard region year-round.

The Svalbard archipelago is a hotspot for many marine mammal species, as well as coastal areas of northeast Greenland and in the marginal ice zone of the Greenland and northern Barents Seas^29^. The Svalbard archipelago is situated at the confluence of the Greenland, Norwegian, and Barents Seas, a region heavily influenced by the West Spitsbergen Current, a branch of the North Atlantic Current that transports relatively warm water and seasonal nutrient pulses to the region^30^. Over the last two decades rapid warming and sea ice loss have resulted in the expansion of warm, salty, nutrient-rich Atlantic Water even further northward causing the Atlantification of Arctic waters^31,32^. The observed shifts in fin whales, an Atlantic marine mammal species, coincided with the intrusion of Atlantic Water and associated Atlantic prey into the waters around and within the fjords of Svalbard^23,25,33,34^.

Fin whales produce several low-frequency calls that vary spatially and temporally, including the 20 Hz call, the 130 Hz call, and the downsweep call^35–37^. The 20 Hz calls are short (~ 1 s) downswept (~ 23–18 Hz) pulses^35,36^. The 130 Hz calls are short (~ 0.3 s) upsweeps from 135 to 140 Hz^37–39^. These have also been referred to as a higher frequency component^37,40,41^ or overtones^42^. Downsweep calls are highly irregular (0.3–1 s) chirps from ~ 100 to 30 Hz^35,39^. Originally described as a 40 Hz sound because they were frequently downswept from 75 to 40 Hz^35^, the 40 Hz call^43^ is believed to be a subset of the 100–30 Hz downsweeps. When produced by males in regular sequences, forming stereotyped songs^35,36,44^, especially in winter, the 20 Hz calls serve a reproductive-related function^36,45^. When produced in irregular sequences^44,46^ or as call-counter calls^47^, especially in summer, 20 Hz pulses likely have a different social context other than song, such as establishing and maintaining contact^44,46,47^. The 20 Hz calls and 130 Hz calls are often documented together, but can be produced independently^28,37,48^. Downsweeps are more common in spring and summer, and have been hypothesized to be associated with feeding^43,49,50^. They have also been associated with signaling and social interactions^35,38,51,52^.

The characteristics of fin whale calls and their acoustic behavior make them an excellent candidate for passive acoustic monitoring (PAM) studies^53^. PAM is a non-invasive method useful in the underwater environment where whales are often easier to detect acoustically rather than visually^54^. It is even more advantageous for long-term monitoring in remote areas where visual methods are not possible or cost-prohibitive^55^. PAM can operate independent of time of day or year, under many different weather conditions, and under full visibility, low-, and no-light conditions. The low-frequency high-intensity characteristic of fin whale calls^56^ increases the area over which they can be monitored^52^ (tens of kilometers) compared to visual methods. Finally, the unique calls made by fin whales allow them to be identified unambiguously.

**Fig. 1:**
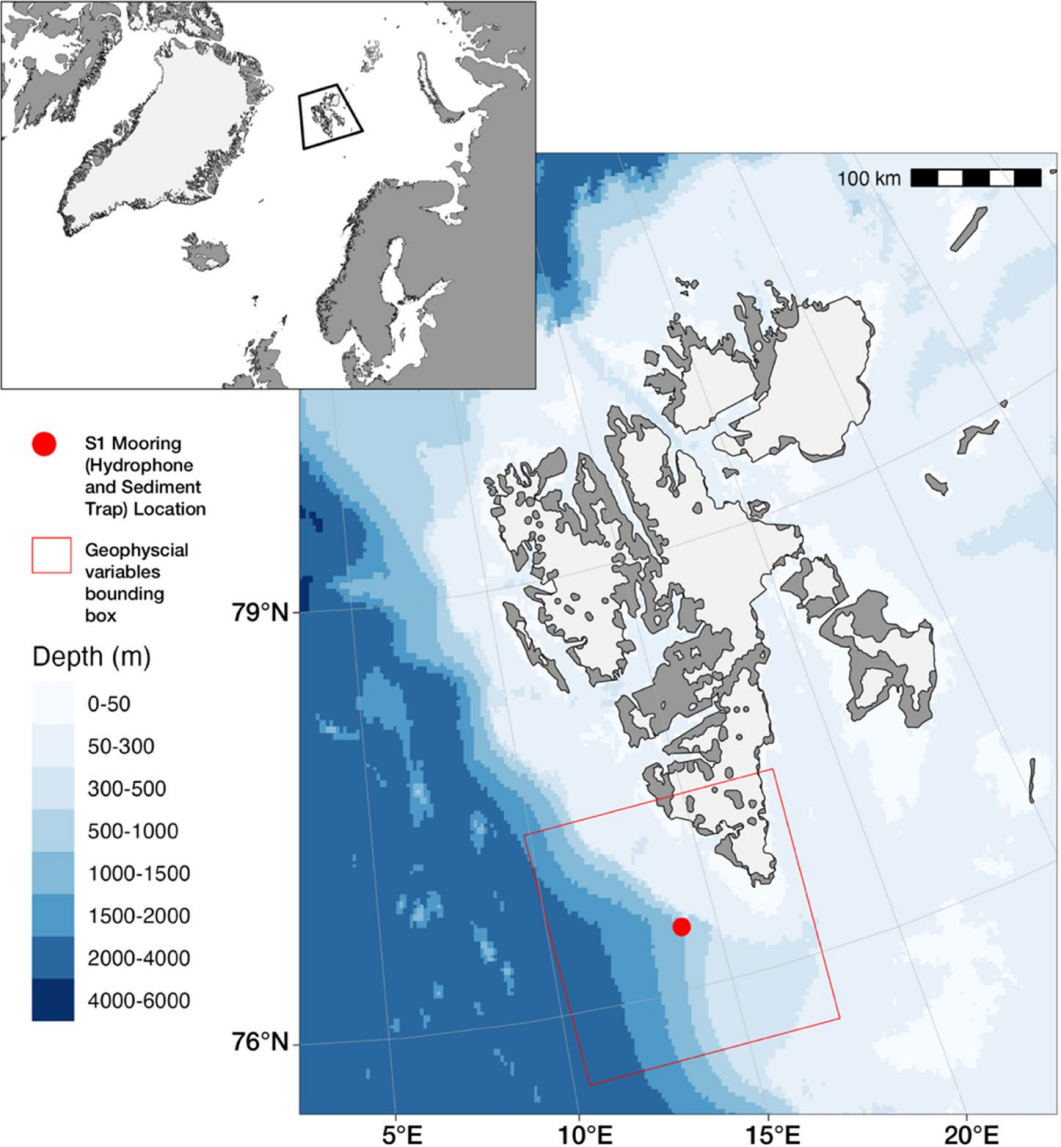
Map and bathymetry of the Svalbard Archipelago. Red circle indicates the S1 mooring location off Southwest Svalbard. Red box indicates where the 200 km x 200 km area where the biogeochemical data were downloaded from the Copernicus Global Ocean Biogeochemistry Analysis and Forecast. Figure made in RStudio (v. 2025.5.1.513)^57^ with R (v. 4.5.1)^58^ using the packages “ggOceanMaps” (v. 2.2.0)^59^, “ggplot2” (v. 3.5.2)^60^, “sf” (v. 1.0–21)^61,62^, and “ggspatial” (v. 1.1.10)^63^.

While fin whale visual and acoustic presence has been documented in the high Arctic, we aimed to quantify fin whale call occurrence in the southwest of Svalbard along the shelf edge where, based on available research, no acoustic studies of fin whale presence have been conducted. With fin whales now being documented in the high Arctic (≥ 80°N), this suggests that this northward expansion also increases their presence in lower latitude Arctic regions. Here, we report on the year-round acoustic presence of fin whales off southwest Svalbard obtained by an acoustic mooring from a deep-sea marine observatory (Fig. 1) and examine the association of their different call types with sea ice, biogeochemistry, and sediment trap data as proxies for local productivity.

## Results

### Fin Whale monthly acoustic presence

**Fig. 2:**
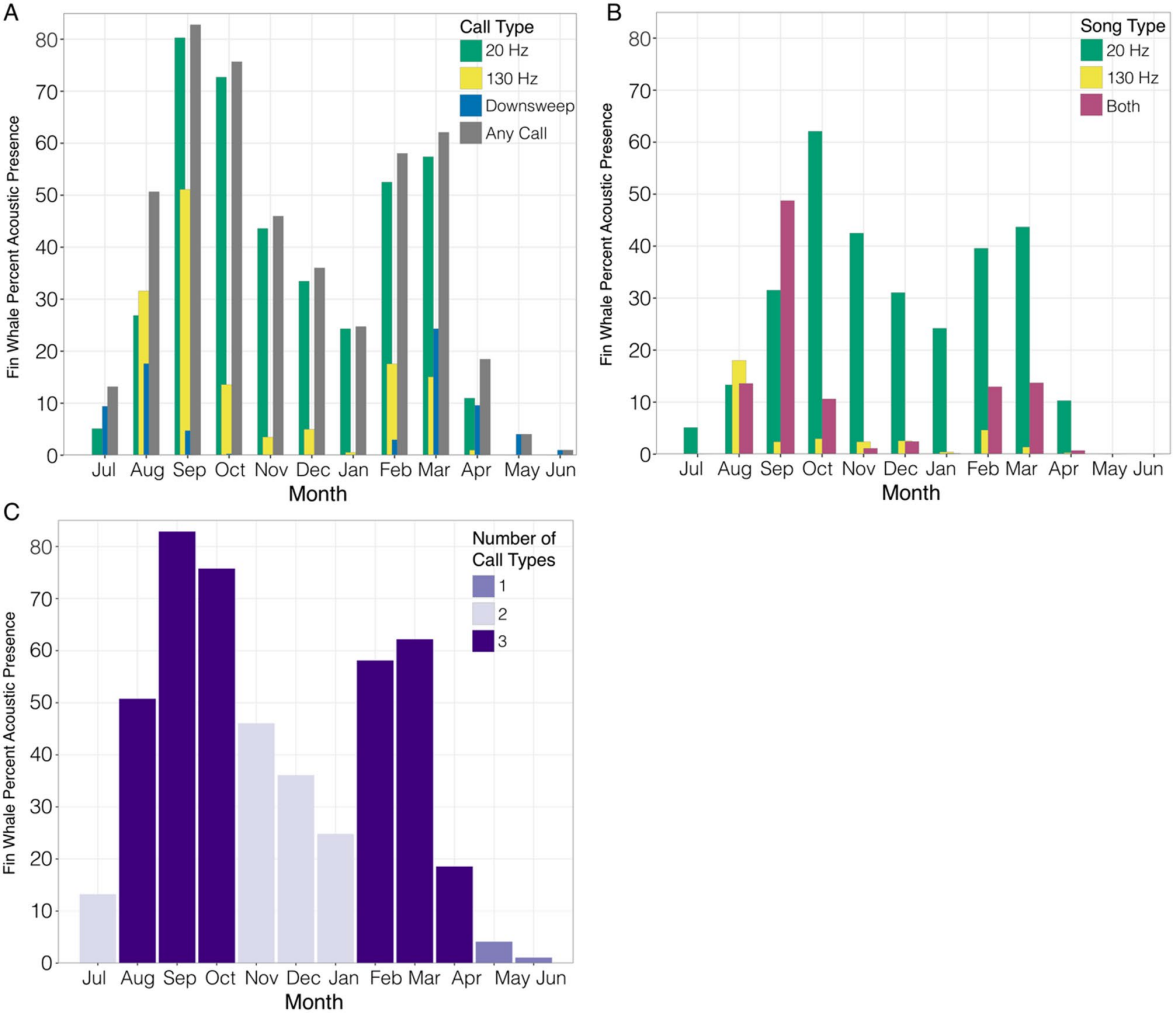
Monthly fin whale percent acoustic presence for (**A**) each call type, including 20 Hz calls (green bars), 130 Hz calls (yellow bars), downsweeps (blue bars), and any call type (gray bars); (**B**) song, including only 20 Hz calls (green bars), only 130 Hz calls (yellow bars), and both 20 Hz and 130 Hz calls in unison (purple bars); and (**C**) number of different call types present per month. Monthly percent acoustic presence was calculated as the number of hours with calls compared to the number of effort hours per month.

Data collection during the Nordic Recognized Environmental Picture (NREP22)^64^ research cruise resulted in ~ 1,140 effort hours from 34,208 2-min acoustic files spanning July 2022 to June 2023 with approximately equal and consistent effort (Table 1). Fin whale calls were detected year-round southwest of the Svalbard Archipelago (Table 1; Fig. 2A–B). This included detections of at least one call type every month and in all but three weeks of deployment effort. Fin whale calls were present for 241 days (65%) out of 358 days of effort. Even with the year-round acoustic presence, seasonal trends were evident depending on call type.

**Table 1:** Monthly detections of fin Whale 20 Hz calls, 130 Hz calls, downsweeps, and any call type, including the number of files, effort hours, call hours, and monthly percent acoustic presence, calculated as number of hours with calls versus number of effort hours per month.

				20 Hz		130 Hz		Downsweep		Any call		
Year	Month	Files	Effort Hr	Call Hr	Pres	Call Hr	Pres	Call Hr	Pres	Call Hr	Pres	Percent Effort
2022	7	2,976	744	38	0.05	0	0.00	70	0.09	98	0.13	13.34
2022	8	2,976	744	200	0.27	235	0.32	131	0.18	377	0.51	13.34
2022	9	2,880	720	578	0.80	368	0.51	34	0.05	596	0.83	13.34
2022	10	2,976	744	541	0.73	101	0.14	2	0.00	563	0.76	13.34
2022	11	2,880	720	314	0.44	25	0.03	0	0.00	331	0.46	13.34
2022	12	2,976	744	249	0.33	37	0.05	0	0.00	268	0.36	13.34
2023	1	2,976	744	181	0.24	4	0.01	0	0.00	184	0.25	13.34
2023	2	2,688	672	353	0.53	118	0.18	20	0.03	390	0.58	13.34
2023	3	2,976	744	427	0.57	112	0.15	181	0.24	462	0.62	13.34
2023	4	2,880	720	79	0.11	7	0.01	69	0.10	133	0.18	13.34
2023	5	2,976	744	0	0.00	0	0.00	30	0.04	30	0.04	13.34
2023	6	2,048	512	0	0.00	0	0.00	5	0.01	5	0.01	9.49

Fin whale 20 Hz calls were detected every month from July through April (Table 1; Fig. 2A, Supplementary Fig. 1) and were only absent for two months from the end of spring into early summer (May–June). The calls displayed a bimodal distribution peaking in September (80%) and October (73%) and again in February (53%) and March (57%). The lowest monthly acoustic presence occurred in July (5%), when the calls were irregular in time and frequency, and April (11%), when many calls were faint.

Fin whale 130 Hz calls were present every month from August to April (Table 1; Fig. 2A, Supplementary Fig. 1) and were absent in July 2022 and May–June 2023. The calls also displayed a bimodal distribution with peaks in August–September and February–March. The greatest monthly acoustic presence occurred in September (51%) and the lowest monthly acoustic presence occurred in January and April (both 1% monthly acoustic presence). Although there were fewer files with 130 Hz calls, the presence patterns for both 20 Hz and 130 Hz calls followed similar trends, with the majority of acoustic presence occurring in late summer and fall, with a secondary, smaller peak in presence from late winter to early spring. Chorusing (overlapping fin whale 20 Hz calls that result in a continuous noise band) with 130 Hz calls occurred in September, November, and December.

Fin whale 20 Hz and 130 calls were detected separately and in unison throughout the timeseries (Fig. 2B). The 20 Hz calls were detected more often than the 130 Hz alone or the two call types together. The trend for the 20 Hz calls alone and both calls in unison were similar, with peaks in September and October and again in February and March. Aside from a peak in August, the 130 Hz calls alone were detected at much lower levels across the year.

Fin whale downsweeps had a bimodal acoustic presence from July through October and February through June (Table 1; Fig. 2A, Supplementary Fig. 1). The downsweeps were completely absent from late-fall through mid-winter (November–January). The calls peaked in August (18%) and March (24%) and the lowest monthly acoustic presence occurred in October and June (where there were a few scattered calls).

The number of call types followed a similar pattern (Table 1; Fig. 2C). Fin whale acoustic presence and number of call types increased in summer from July to August. Acoustic presence peaked in September and began to decline in late fall and into winter (November–January). In February, call type and presence increased again and peaked in March before declining sharply from March to April. Fall and spring had the highest number of fin whale call types and presence and the period from late spring through summer had the lowest.

### Diel trends in acoustic presence

**Fig. 3:**
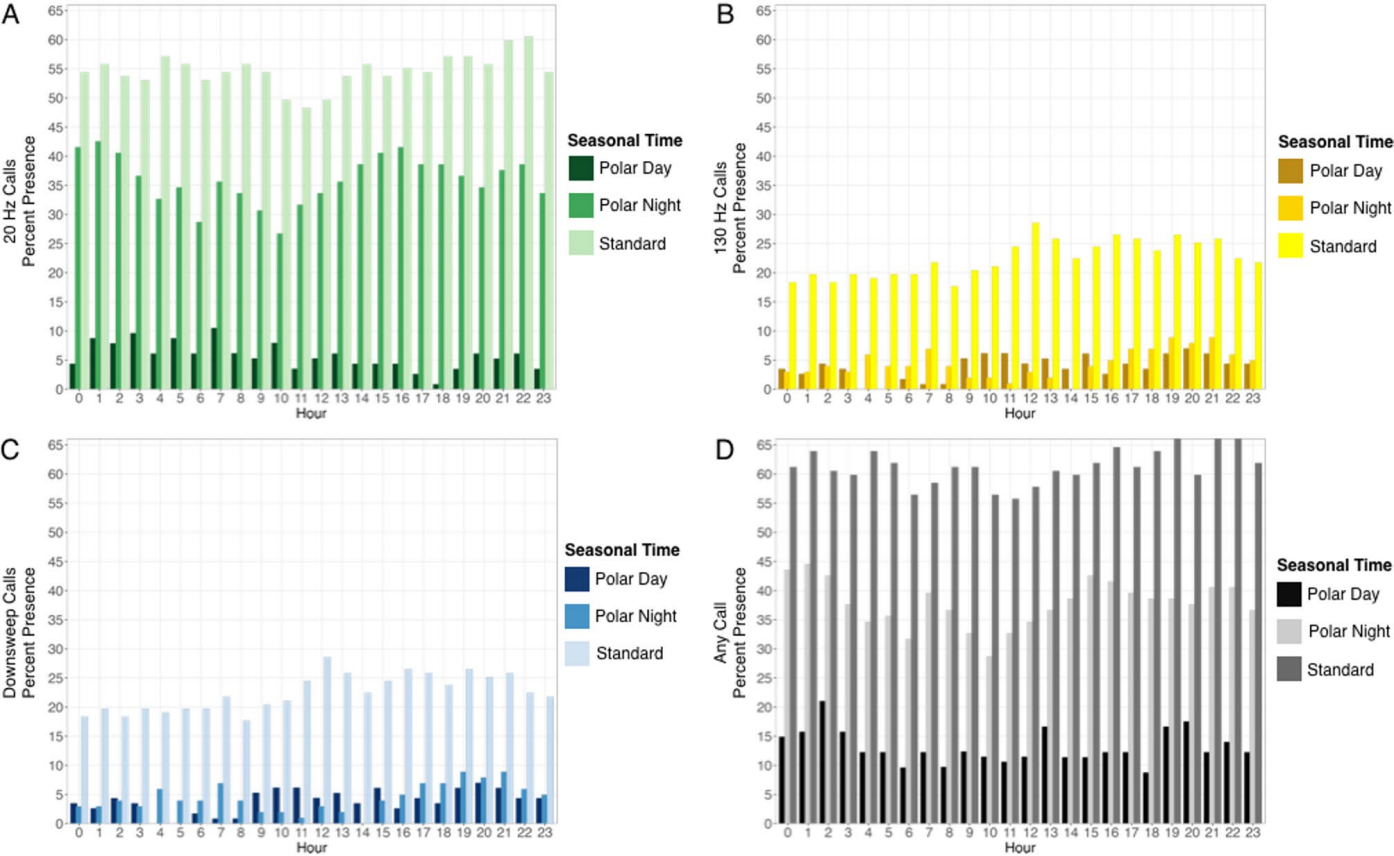
Diel trends in hourly fin whale percent acoustic presence for (**A**) 20 Hz calls (green bars), (**B**) 130 Hz calls (yellow bars), (**C**) downsweeps (blue bars), and (D) any call type (gray bars). The color gradients indicate the different solar periods throughout the year (i.e., polar day, polar night, and the standard solar cycle).

No strong diel trends were evident, even when the solar cycle was accounted for, i.e., standard season, polar night (complete darkness), which spanned December to January, or polar day (where the sun never sets), which spanned the end of April to mid-August (Fig. 3A-D). The presence of 20 Hz calls slightly dipped midday during polar night and the standard solar cycle, but the trend was not the same during polar day. There were slightly more 130 Hz calls at night during the standard solar cycle, and calls peaked after midnight and in the early afternoon during polar night, but there were no obvious trends during polar day. There was a slight increase in the presence of downsweeps after midday during the standard solar cycle and a reduction in calls midday during polar night, however there was no clear trend during polar day.

### Biotic and abiotic data

**Fig. 4:**
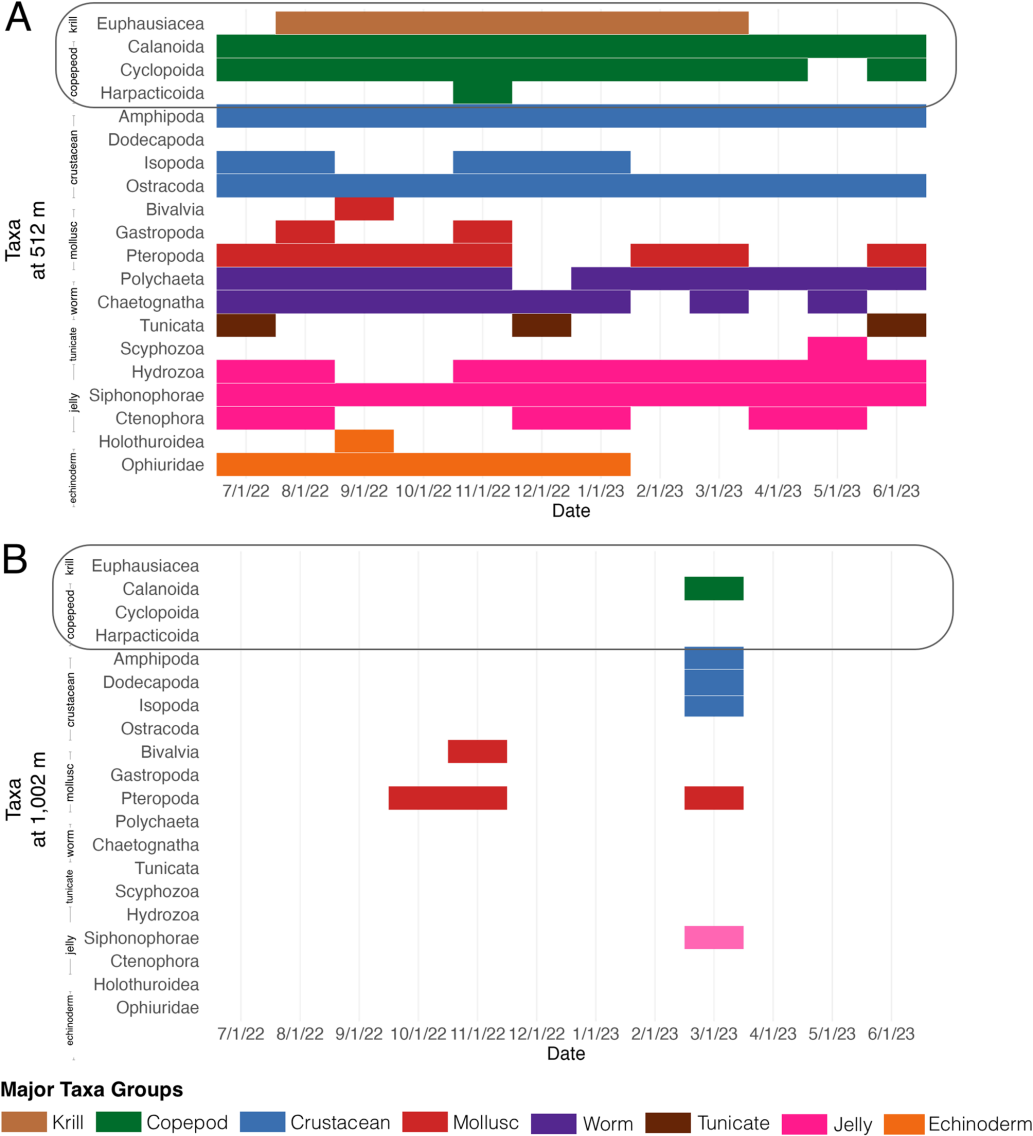
Major taxa identified in the sediment traps at (**A**) 512 m and (**B**) 1,002 m from July 2022 through June 2023 at the S1 mooring southwest of Svalbard. Taxa are grouped and colored by broad categories (i.e., krill, copepod, crustacean, mollusc, worm, tunicate, jelly, echinoderm) for visual purposes. The first four taxa (enclosed) represent major taxa that may be prey for fin whales.

The sediment trap at 516 m captured samples each month ranging from 107 taxa to 470 taxa Table 2). There appeared to be three time periods with high numbers of taxa caught in the sediment trap: September (316 taxa), February (470 taxa), and May (467 taxa) (Table 2; Fig. 4). Between July and September 2022, six genera of copepods were sampled, including the carnivorous *Heterorhabdus norvegicus* (*n* = 10), deep-sea predators of the genus *Paraeuchaeta* spp. (*n* = 9), and *Gaetanus* spp. (*n* = 5). Cyclopoid copepods (*n* = 236) and ostracods of the family *Halocyprididae* (*n* = 190) dominated until October 2022, whereas gelatinous taxa began appearing in high abundances, including *Siphonophorae* (*n* = 60) and *Chaetognatha* (*n* = 10), until December 2022. In January 2023, gelatinous zooplankton was highly represented (four taxonomic units), along with the isopod *Gnathia maxillaris* (*n* = 7) and pelagic amphipods of the genus *Themisto* spp. (*n* = 21). Between February and April, the pteropod *Limacina retroversa* (*n* = 134) and *Halocyprididae* (*n* = 293) increased in abundance. Additionally, juvenile *Themisto* spp. was collected (*n* = 454) between April and May 2023. *Cyclocaris* spp. appeared in July 2022 (*n* = 1) and February 2023 (*n* = 1). Nearly half of all samples were comprised of prey relevant to fin whales, including *Euphausiacea* (*n* = 16) that were present between August 2022 and March 2023; copepods (*n* = 866; i.e., *Cyclopoida*, *Harpacticoida*, *Calanoida*) and amphipods that were present between July 2022 and June 2023 (*n* = 545).

**Table 2:** Monthly biotic and abiotic variables assessed relative to fin Whale acoustic presence, including total abundance of zooplankton collected with the two sediment traps at 516 m and 1,002 m; mean concentration of zooplankton biomass (mmol/m^3^), phytoplankton biomass (mmol/m^3^), net primary production of biomass (mg/m^3^/day), and chlorophyll a (mg/m^3^) in the 200 km x 200 km area around the hydrophone; and mean sea ice area (km^2^) and extent (km^2^) in the Greenland Sea.

Year	Month	Taxa (516-m)	Taxa (1,002-m)	Zooplankton Biomass	Phytoplankton Biomass	Net Primary Production	Chlorophyll a	Sea ice area	Sea ice extent
2022	7	181	0	5.61	10.82	19.33	1.65	220,052.30	376,029.27
2022	8	193	0	1.15	3.53	15.48	0.63	165,321.65	278,536.57
2022	9	316	0	0.44	3.90	12.63	0.81	160,912.54	285,351.52
2022	10	224	20	0.002	1.17	1.30	0.27	255,500.49	365,256.14
2022	11	168	37	0.0002	0.27	0.0	0.067	364,573.07	488,293.21
2022	12	129	0	0.002	0.073	0.0	0.013	451,148.13	635,073.90
2023	1	140	0	0.002	0.025	0.0	0.004	509,900.17	684,651.89
2023	2	470	0	0.002	0.017	0.0	0.003	520,020.26	698,053.28
2023	3	178	29	0.002	0.017	0.008	0.003	645,716.30	846,211.94
2023	4	378	0	0.002	0.083	0.19	0.015	631,264.19	831,596.37
2023	5	467	0	0.004	6.88	28.71	1.056	597,400.91	834,849.72
2023	6	107	0	6.00	15.30	35.77	2.40	452,333.57	721,350.52

At 1,002 m, the sediment trap captured samples only in October-November 2022 and March 2023 (Table 2; Fig. 4). In October 2022, only the planktonic gastropod *Limacina retroversa* (*n* = 20) was collected. In November 2022, 37 specimens were retrieved, comprising 36 samples of *Limacina retroversa* and one aquatic mollusc (Class Bivalvia). In March 2023, 29 samples were gathered, representing 9 different taxa groups. The majority of samples were copepods, including Genus *Paraeuchaeta* (*n* = 7) and their nauplii (*n* = 3), Genus *Calanus* (*n* = 5), and Genus *Aetideopsis* (*n* = 3). Other taxa included the amphipod *Cyclocaris guilelmi* (*n* = 4), and the *Limacina helicina* (*n* = 3) and *Limacina retroversa* (*n* = 1); one pelagic shrimp (Genus *Hymenodora*); one isopod (Order *Isopoda*); and one siphonophore (Order *Siphonophorae*).

The concentration of zooplankton and phytoplankton biomass, net primary production of biomass, and chlorophyll-*a* in the 200 km x 200 km area around the hydrophone displayed similar trends (Table 2; Supplementary Fig. 2). Mean zooplankton biomass decreased by 92% from July (5.61 mmol/m^3^) to September 2022 (0.44 mmol/m^3^). Biomass remained low (< 0.002 mmol/m^3^) until May 2023 then reaching its maximum in June 2023 (6.00 mmol/m^3^). Similarly, mean phytoplankton biomass decreased by 97% from July 2022 (10.82 mmol/m^3^) to November 2022 (0.27 mmol/m^3^). Concentration remained < 0.08 mmol/m^3^ until increasing again in May 2023 and peaking in June 2023 (15.30 mmol/m^3^). Mean net primary production biomass in June (19.33 mmol/m^3^/day) decreased 93% by October 2022. Concentration remained at zero until March 2023 then began increasing to its peak (35.77 mg/m^3^/day) in June 2023. Mean chlorophyll *a* biomass decreased 84% from June (1.65 mg/m^3^) to October 2022 (0.27 mg/m^3^). Concentration remained < 0.1 mg/m^3^ increasing to its peak (2.4 mmol/m^3^) in June 2023. Sea ice area and extent was at its lowest in August and September 2022 (160,912 km^2^ and 285,351 km^2^, respectively) and peaked (645,716 km^2^ and 846,211 km^2^, respectively) in March 2023 (Table 2).

### Monthly models

**Fig. 5:**
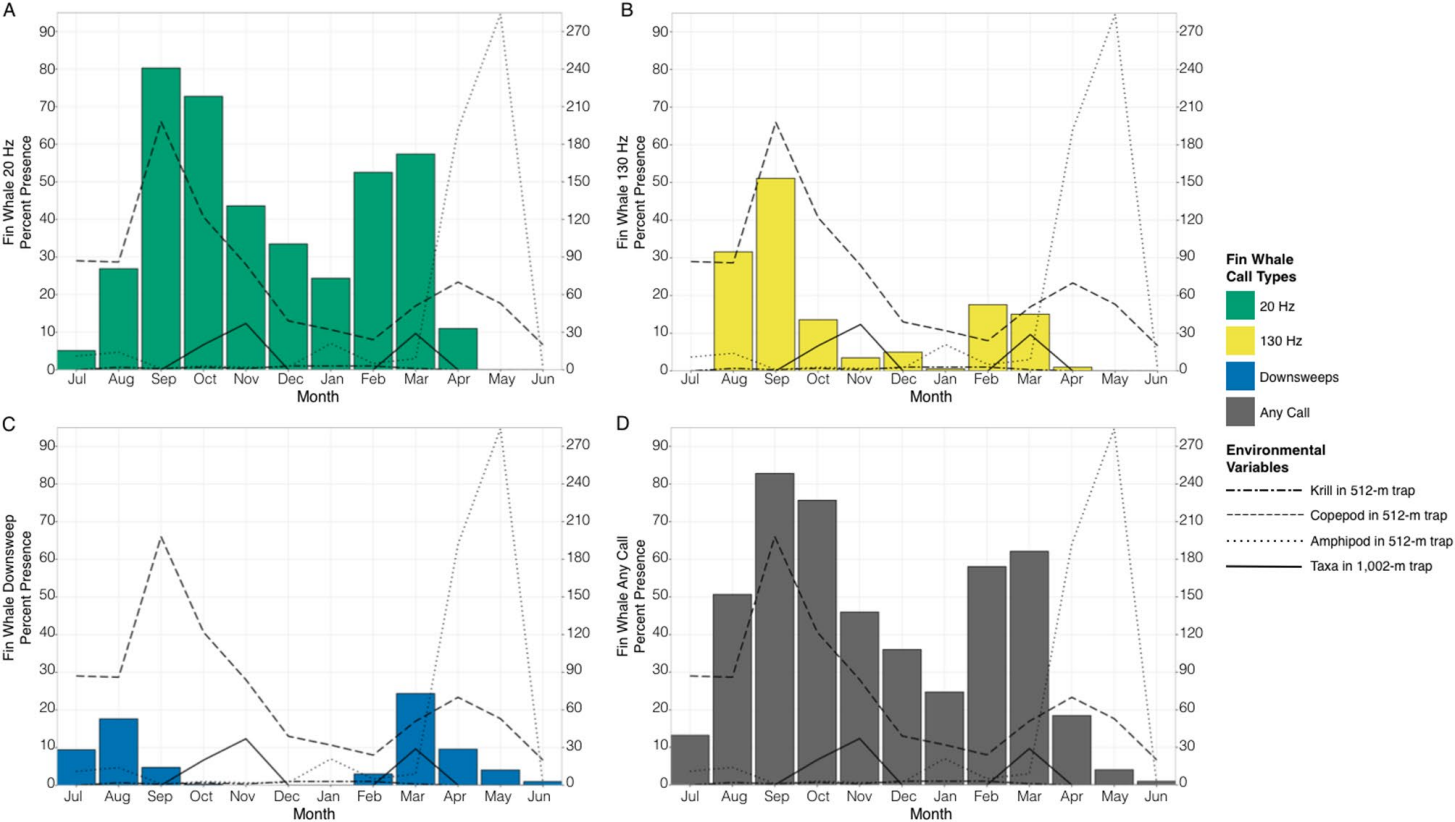
Monthly fin whale percent acoustic presence for (**A**) 20 Hz calls (green bars), (**B**) 130 Hz calls (yellow bars), (**C**) downsweeps (blue bars), and (**D**) any call type (gray bars) compared to monthly number of krill (two-dash black), copepod (dashed black line), and amphipod (dotted black line) from the 516-m sediment trap, and number of taxa in the 1,002-m trap (solid black line). Data span from July 2022 to June 2023.

The majority of the monthly abiotic and biotic variables were more than moderately correlated (> 0.50 r; Supplementary Fig. 3). As a result, only number of taxa in the 1,002-m sediment trap, and number of krill, copepods, and amphipods in the 516-m sediment trap were included for modeling the temporal relationships with each call type and all call types combined (Fig. 5A-D; Table 3, Supplementary Fig. 4, Supplementary Table 1).

**Table 3:** Summary of model outputs from best fitting models retained in model selection for 20 Hz calls, 130 Hz calls, downsweeps, and any call type.

Call	Predictors	Estimated std. Error		CI	Statistic	*p*
20 Hz	(Intercept)	-12.41	13.16	-38.20–13.38	-0.94	0.346
	1000-m Trap	0.79	0.36	0.09–1.49	2.20	**0.028**
	Krill	12.35	4.45	3.62–21.08	2.77	**0.006**
	Copepods	0.35	0.1	0.16–0.54	3.34	**< 0.001**
	Amphipod	-0.02	0.06	-0.14–0.10	-0.31	0.753
	Observations	12				
	R^2^	0.81				
130 Hz	(Intercept)	-12.13	7.11	-26.06–1.80	-1.71	0.088
	Krill	4.43	2.66	-0.79–9.65	1.66	0.096
	Copepods	0.25	0.07	0.12–0.38	3.76	**< 0.001**
	Observations	12				
	R^2^	0.63				
Downsweep	(Intercept)	8.14	3.48	1.32–14.97	2.34	**0.019**
	krill	-1.49	1.96	-5.32–2.35	-0.76	0.447
	Observations	12				
	R^2^	0.06				
Any Call	(Intercept)	-10.18	9.24	-28.29–7.94	-1.10	0.271
	1000-m Trap	0.72	0.31	0.12–1.33	2.34	**0.019**
	Krill	13.24	3.4	6.57–19.91	3.89	**< 0.001**
	Copepods	0.37	0.08	0.21–0.54	4.4	**< 0.001**
	Observations	12				
	R^2^	0.82				

The bold values indicate statistical significance.

Model selection using the monthly acoustic presence of 20 Hz calls retained all the variables (R^2^ = 0.811) and found a significant positive relationship with number of taxa in the 1,002-m sediment trap (*p* = 0.028), krill (*p* = 0.006) and copepod (*p* < 0.001) in the 516-m sediment trap (Table 3, Supplementary Table 1). The Shapiro-Wilk normality test and Durbin Watson Test were not significant, indicating a normal distribution and non-correlated residuals and the Variance Inflation Factors (VIF) suggested no collinearity (Supplementary Fig. 4).

Model selection using the monthly presence of 130 Hz calls retained krill and copepod (R^2^ = 0.63) in the 516 m sediment trap and found a significant positive relationship with copepods (*p* < 0.01) and a positive relationship with krill (*p* = 0.096) in the 516-m sediment trap (Table 3, Supplementary Table 1). The Shapiro-Wilk normality test and Durbin Watson Test were not significant, indicating a normal distribution and non-correlated residuals and the VIF suggested no collinearity (Supplementary Fig. 4).

Model selection using the monthly acoustic presence of downsweeps retained krill but did not result in a significant relationship (Table 3, Supplementary Table 1).

Model selection using the monthly acoustic presence of any call type retained number of taxa in the 1,002-m sediment trap and number of krill and copepods in the 512-m sediment trap (R^2^ = 0.50). All three were significantly positively correlated (*p* < 0.02) with the monthly acoustic presence of any call type (Table 3, Supplementary Table 1). The Shapiro-Wilk normality test and Durbin Watson Test were not significant, indicating a normal distribution and non-correlated residuals and the VIF suggested no collinearity (Supplementary Fig. 4).

## Discussion

### Temporal presence and context of calls

Our study reveals that fin whales were acoustically present off southwest Svalbard year-round from July 2022 to June 2023. The 20 Hz and 130 Hz calls peaked in fall and spring and were absent in summer, whereas the downsweeps were bimodally present during both spring and fall. The year-round presence of the three different call types suggests this habitat is a mixed-use area for foraging and reproductive-related activities based on the suggested behavioral functions of the call types^36,43,45,49,50^.

The trend through the entire North Atlantic has generally been described as an increase in song in fall, a peak in winter, and a decrease in spring^49,65^. Our study found 20 Hz calls were present July–April and 130 Hz were present August–April. The two were only absent simultaneously for two months in May and June, with both peaking in September. This is slightly earlier than the November–December peak documented by in Davis Strait^37^. However, Davis Strait is thousands of km west and nearly 10° lower in latitude than our site.

The timing and peak of our 20 Hz and 130 Hz calls are similar to ranges documented in regions closer to our study site. For example, while fin whales were documented intermittently with no seasonal or temporal pattern in western Fram Strait, in Central Fram Strait (~ 400 km west and 2° north of our study site), fin whales were present from July through December, with peak occurrences in August and October^24^. In Kongsfjorden, a fjord in northwest Svalbard (~ 290 km and 3° north of our site), 20 Hz and 130 Hz calls were detected year-round with a higher detection rate starting in October at the onset of polar night^28^. Finally, on the continental shelf north of the Svalbard Archipelago (> 700 km and 5° north of our study site), Ahonen et al. (2021)^24^ documented calls seasonally from mid-September through until early November with no calls December to July.

It is worth noting that there was also significant noise in low frequency (< 150 Hz) bands, which resulted in high levels of masking, especially in November but also December–January. The high levels of noise corresponded with a decrease in the monthly acoustic presence of 20 Hz calls November–January. This may have masked a later winter peak in 20 Hz calls and could also explain the apparent second peak February-March. Additionally, we observed a decrease in the monthly acoustic presence of 130 Hz calls from November through January. Despite fewer overall detections of 130 Hz calls, the seasonal pattern of the 20 Hz calls and the combination of the 20 Hz and 130 Hz calls followed a similar trend. This is supported by previous studies in the North Atlantic^28,37,48^ and other regions^53,66^ that showed 20 Hz and 130 Hz calls produced in unison.

That being said, Papale et al. (2023)^28^ only documented 30% of songs made up of 20 Hz and 130 Hz notes simultaneously, which is similar to the 23% we estimate from our study. They also documented both calls independently, leaving the role of 130 Hz calls when produced with or independent of 20 Hz calls (i.e., song versus long-range communication) unknown. Simon et al. (2010)^37^ noted that the high frequency components are more directional and thus only recorded when the whale is pointing in the direction of the recorders. This could also explain why there were fewer 130 Hz calls detected alone or in unison with the 20 Hz calls.

Interestingly there were no strong diel trends in calling for any call type and regardless of solar cycle (i.e., standard season, polar night, and polar day). Simon et al. (2010)^37^ documented singing strongly linked to daylight hours, with singing starting in the afternoon and last through the evening. The timing shifted with the season, getting progressively earlier and ending later indicating of feeding during daylight hours. But as we noted, this was a site father away and lower in latitude than our study and may include other fin whale populations where migratory behavior may be different. However, Ahonen et al. (2021)^24^ noted loud fin whale signals occurred in bouts that lasted from hours to a few days, suggesting there may be less of a diel difference at some of these higher latitude sites.

Compared to the sediment trap at 1,002 m, the intermediate-depth trap at 516 m revealed a greater diversity of taxa, with notable seasonal shifts in species composition and abundance. This suggests that different depth layers support distinct communities, likely influenced by varying environmental conditions and food availability throughout the year. Bimodal peaks in 20 Hz and 130 Hz calls occurred at the same time as bimodal peaks in copepods in the 516 m sediment trap. *Calanus* spp. and *Metridia longa* were present in high numbers and are important prey for fin whales^67^. Krill in the 516 m sediment trap remained low the entire year, but was higher prior to peaks 20 HZ and 130 Hz calls, with krill dropping to lower numbers during and after the peaks. While a high number of copepods suggest they could be a potential prey source for fin whales, the krill samples in the sediment trap were all identified as *Thysanoessa inermis*, which is also an important prey species for fin whales^68^.

There were also more 20 Hz calls when amphipods in the 516 m sediment trap were elevated. The highest amphipod counts occurred at the end of May and into June, which is when all call types significantly decreased. This suggests that fin whales were not targeting amphipods and that they likely left the area for other more productive regions, such as the fjords where *T. inermis* dominate^69^, or were potentially engaged in mating. However, fin whales also feed on fish; therefore, without information on fish in the region, prey selection during this study remains unknown.

The exact locations of fin whale calving, mating, and wintering remain unknown^70,71^. Breeding is estimated to occur in winter (November–February in the Northern Hemisphere), with peak conception in January^72,73^. Birth is estimated to occur the following year in wintering grounds^14^. Simon et al. (2010)^37^ noted that peaks in song in Davis Strait overlapped with the conception period (November–December), and suggested whales may mate at high latitudes. Our study confirms that fin whales are present off Svalbard during the estimated conception period, despite a decrease in calling compared to the fall. While our data do not allow for an estimate of whale abundance, the presence of heavy chorusing (overlapping 20 Hz and 130 Hz calls) during this time (especially September, November, and December) suggests the presence of several individuals singing at the same time. If song serves as a mating display, possibly to attract mates^36,45^, then the fall peak we documented was likely related to mating, with calls declining after copulation occurred.

In summer, 20 Hz calls are believed to serve a different, non-song-related function^37,44,46,47^. While we detected faint and irregular 20 Hz calls in spring and early summer, 20 Hz and 130 Hz calls were largely absent in the summer. Similarly, Romagosa et al. (2021)^49^ reported the absence of 20 Hz calls in summer. This may indicate that whales have migrated farther offshore; however, the presence of downsweeps suggests that some fin whales remained in the region, albeit in a different behavioral state. It is likely that fin whales shifted to foraging during this period, which would explain the shift to downsweeps.

Downsweeps are frequently documented in the spring and summer^35,43^. The downsweeps detected in our study were present in spring through fall and absent in the winter, with peaks in March and August. If the calls are related to foraging activity^43,49,50^, then these peaks likely correspond to peak feeding periods. Their complete absence from fall through winter suggests that by then whales have shifted into reproduction-related behaviors. Romagosa et al. (2021)^49^ found that zooplankton biomass peaks in spring and fall had a significant effect on downsweep call production. However, the downsweeps showed no correlation with any of the sediment trap data we included in the models. It is unclear how well a sediment trap at one location matches the entire acoustic range or if whales removed potential prey before it reached the sediment traps. That being said they may better match the peaks of the broad-scale mean monthly zooplankton, phytoplankton, net primary production, and chlorophyll a biomass data, which occurred in spring and summer when the downsweeps were present. Fin whales have also been associated with euphausiids^74^, capelin^74^, cod^75^ and herring^76,77^ around Svalbard. Even with more detailed prey data, ecological relationships are often weak, and even when prey is sampled concurrently, it is not always the best predictor of whale abundance^78,79^.

### Importance of including multiple call types

No other study to-date has reported on such a high degree year-round presence. Some have inferred year-round presence based on the months that fin whales were detected^24,27^. Some of these gaps in fin whale presence could be explained by the detection method used and/or the call types these studies looked for. Other studies in the region used cross-correlation algorithms to detect individual calls typically focused on 20 Hz calls^20,24,27^. Although Meister et al. (2024)^20^ employed human analysts to manually detect 130 Hz calls on an hourly basis. Studies using energy detectors did not report year-round presence of fin whales and generally only assessed energy around the 20 Hz band^28,80–82^. In both cases, gaps in fin whale detection align with the seasonality of fin whale 20 Hz calls. The presence or absence of calls would also depend on the hydrophone(s) location relative to high fin whale core use areas.

Additionally, the methods for detecting fin whales have limitations. For example, energy detectors can detect fin whales from hundreds to thousands of km away, but may struggle to distinguish them from similar signals, like air guns. Algorithms using cross-correlation miss fin whale presence during intense chorusing. In our tests, both energy detectors and call cross-correlation algorithms performed poorly compared to human analysts. The cross-correlation algorithms were ineffective during chorusing periods while strumming and higher noise floors reduced the accuracy of the energy detectors. In some cases, the presence of the 130 Hz calls during high noise levels was the only reliable indicator of fin whale presence.

Manual analysis provided a more accurate assessment of presence/absence for this study. It also allowed for the annotation of multiple call types occurring at different times of the year, reflecting distinct behavioral contexts^43^ and offering greater insight into their seasonal patterns. However, manual analysis of multiple years of data is time-consuming, subject to potential bias, and not always feasible^83,84^. A future approach combining cross-correlation algorithms, energy detectors and human verification could provide a more comprehensive understanding of fin whale ecology.

### Migration revisited

Given the ongoing discoveries about baleen whale migration patterns, it is unsurprising that the North Atlantic fin whales deviate from the typical migratory patterns. Geijer et al. (2016)^6^ identified only three fin whale populations that were migratory, four were non-migratory, five were short-range migrants, and two others two exhibited both migratory and non-migratory behaviors. Although the Northeast Pacific population was classified as migratory^85,86^, a resident sub-population in southern California has been documented^87,88^. Similarly, while the larger North Atlantic fin whale population is often considered migratory, a smaller subset of the population, or potential sub-population, off Svalbard might be non-migratory. The question is why they are remaining year-round.

One possible explanation is the reduction of sea ice. Historically, the northern summer feeding range of fin whales was constrained by the ice edge^70,89^. Simon et al. (2010)^37^ found a negative correlation between sea ice extent and fin whale distribution, reinforcing the idea that sea ice limited their movement northward. However, sea ice is rapidly declining in the Arctic, with the most significant losses occurring in the Barents Sea^90^. As sea ice extent shrinks, it may result in shifts in species composition, abundance, and distribution^91^, which would create suitable habitats for fin whales^92^ and other whale species^93^.

Fin whales are now documented as far north as 81.5° N^23,24^. Similar trends have been observed in the Pacific, where the fin whales are recently detected off Utqiaġvik, the northernmost record ever documented in the Alaskan Arctic^94^. North Atlantic right whales (*Eubalaena glacialis*) also returned to historically important areas such as the southern New England shelf, a repatriation (i.e., a return to historic distributions) likely driven by climate-driven changes in the abundance and distribution of the North Atlantic right whale’s primary prey species^12^. These patterns suggest that the loss of sea ice is driving, in part, the increased abundance of fin whales off Svalbard and their northward expansion in the high Arctic.

Another possibility is that there is an expansion of recovering populations following a 50-year cessation of commercial whaling. Fin whales were hunted by Norwegian whalers from Bear Island and northwards, including areas west of Svalbard. Spitsbergen operated floating whaling factories until 1909^95^. Therefore, their presence in this region may not be a new phenomenon. Repopulation of historical whaling grounds is occurring around the world. Fin whales have returned to historic whaling grounds in the Southern Ocean^13^. Blue whales (*Balaenoptera musculus*), southern right whales (*Eubalaena australis*), and humpback whales (*Megaptera novaeangliae*) have been documented returning to a feeding ground and former whaling area off South Georgia Island in sub-Antarctic waters after 4–5 decades^11,96,97^. Southern right and humpback whales have also returned to calving areas that were former whaling areas in Australia and New Zealand^98,99^.

Fin whale abundance in the Northeast Atlantic (spanning the North Sea to the ice edge of the Barents/Greenland Seas) was estimated at 11,387 individuals (95% CI: 8,072–16,063) from sighting surveys in 2014–2018^100^. The population was estimated to be increasing at a rate of 4% per year^21^. Thus, the increase in populations around the continental shelf-slope areas west and north of Svalbard may have resulted from the expansion of fin whale distribution from their primary habitat around Iceland^101^.

Whales off Svalbard would likely belong to either the north Norway/Arctic Eastern North Atlantic or west Norway/Faroe Islands stocks delineated by the International Whaling Commission^102^. However, some tagged fin whales that spent the summer off Svalbard were observed to migrate to the southwest waters off Portugal in the fall and winter, suggesting mixing occurs between populations^26^.

At the same time potential fin whale prey is increasing off Svalbard, including krill and amphipods in the high Arctic Kongsfjorden, west Spitsbergen^103–105^. Fish species, including mackerel^106^ and cod^107^ are also increasing in Svalbard coastal and fjord waters where fin whales are now frequently observed. Many questions remain about the population structure of fin whales around Svalbard. It is unknown if the same fin whales remain year-round or in the regions or if subpopulations of fin whales are sharing a common feeding grounds within the North Atlantic at different times during the year^108^.

### Gaps and limitations

A few limitations from our study should be noted. First, we only included one year of data in our study. While with this dataset, we can say with certainty that fin whales were present year-round during this time off Svalbard, it would be beneficial to include additional years of data to fully understand the seasonal patterns, especially if they are changing over time. Additionally, there is a loss of granularity from using presence/absence for the call types rather than annotating each individual call. Acoustic absence does not always mean true absence and analysis of presence, while useful, does not provide information on population structure^39,80,109^ or ecology of the population that would shed additional light on the trends we found. A more detailed analysis of calling patterns is needed to reveal those elements. Because the hydrophone recorded on a duty cycle (i.e., sampling 2 min every 13 min), this may have resulted in missed vocalizations, especially more intermittent call types such as downsweeps. This could result in potentially different peaks in presence than reported. As additional limitation of this study was the lack of information on fish species in the region, which would have provided additional evidence for presence and prey selection. Finally, to extract the biogeochemistry data for our analyses we used a nominally estimate that fin whale detections do not extend beyond 100 km in any direction. Although we did not estimate detection ranges for this study, similar studies have reported 85 km in Davis Strait^37^, 64 km for the 20 Hz call and 18 km for the downsweep call in the Northern Atlantic Ocean including Fram Strait^49^, and 55 km in deep water in Fram Strait and 30 km near the shelf^24^. Thus, the detection range for our study could be similar (33–85 km).

## Conclusion

As Arctic sea ice continues to decline, fin whales are expanding their northern extent into higher latitudes. This also appears to be increasing their presence in lower Arctic regions. From one year of acoustic data collected from a mooring on the continental slope southwest of Svalbard, we were able to investigate the temporal presence of fin whale vocalizations. Detections and call diversity were highest in fall and spring and lowest during summer but fin whales were present year-round. Downsweeps, which may be more indicative of feeding behavior were present spring through fall and absent in winter, when only 20 Hz and 130 Hz calls, indicating of reproductive-related calling, remained. While some of these trends in calling could be explained by the biotic and abiotic data we modeled, the seasonality of calls suggests this habitat is a mixed-use area for foraging and reproductive related activities.

However, key questions remain about the number of animals present southwest of Svalbard and individual residence times in the region and if whales detected throughout the year remained, were replaced by other whales, and/or returned after moving between feeding areas. Additional mooring locations, visual surveys, and satellite tag data would help clarify how many fin whales overwinter off Svalbard and whether they remain year-round or migrate elsewhere in the North Atlantic. Song could be further analyzed to assess inter-note intervals (INIs; the time interval between 20 Hz notes), which could help clarify if whales in the region are from different populations. Biopsies would also allow for genetic testing. Acoustic data indicate that their behavior off Svalbard are related to both feeding and reproduction, but the broader implications of these shifts are still uncertain. As the Arctic warms, it remains unclear how the distribution patterns of fin whale habitats will evolve. Longer-term monitoring is essential to understand the changing structure of fin whale habitats, prey fields, and distributions.

## Methods

Data were collected from the oceanographic mooring S1 (76.44°N, 13.95°E, Fig. 1) deployed by the R/V Alliance during the NREP22^64^ research cruise led by the North Atlantic Treaty Organization (NATO) Science and Technology Organization’s (STO) Centre for Maritime Research and Experimentation (CMRE). The S1 mooring has been deployed at 1,042 m water depth and maintained since 2014 by the National Research Council, Institute of Polar Sciences (CNR-ISP) and the National Institute of Oceanography and Applied Geophysics (OGS), and was initially positioned because its location lies strategically at the convergence of Atlantic Water with dense waters from Storfjorden and shelf waters from the West Spitsbergen continental shelf^110,111^.

### Passive acoustics

The S1 mooring (Supplementary Fig. 5) was equipped with an omnidirectional Autonomous Underwater Acoustic Data Logger (Loggerhead Instruments Inc., Sarasota, FL, USA). Passive acoustic data were collected between 25 June 2022 at 10:30:00 UTC and 22 June 2023 at 06:00:00 UTC (Fig. 1). The hydrophone was located at a depth of ~ 557 m, recording with a sampling frequency of 96 kHz, a system sensitivity of -170 dB re 1 V/µPa, gain of 2 dB re 1 V/1V, bit rate of 16-bit, and A-to-D converter zero-to-peak voltage of 1.0 V. The hydrophone was operated on a duty cycle to sample at 0.13 Hz (2 min on/13 min off), resulting in four files per hour.

### Fin Whale call detection

Because of the combination of whale chorusing and intermittent mooring strumming noise in lower frequencies (< 100 Hz), automatic detectors did not perform well. Detectors designed to isolate individual calls resulted in many false positives and energy detectors provided false results during periods of intense masking in the 20-Hz band. Therefore, a visual analysis using spectrograms was performed in order to isolate the calls. The acoustic data were decimated by a factor of 10 using the Triton software package in Matlab (MathWorks Inc., Natick USA). After decimation, a total of 1,157 h of passive acoustic data was visually examined using Raven Pro 1.6.5 (K. Lisa Yang Center for Conservation Bioacoustics at the Cornell Lab of Ornithology, Ithaca, USA). We used a 960 s window (equivalent to 8 files and two hours with duty cycling), a FFT of 9,000 samples using a Hann window with 50% overlap and with brightness and contrast set to 50%. Each 2-minute file was assessed for the presence of 20 Hz calls, 130 Hz upsweeps, or irregular downsweeps generally between 100 and 30 Hz (Supplementary Fig. 1). Detections with 20 Hz and/or 130 Hz calls were further analyzed to note when each song-associated call type was identified at the same time or independently. Detections were summarized to monthly detections. Monthly percent acoustic presence was calculated as number of hours with calls versus number of effort hours per month. This was calculated for each call type and a combined call variable, which considered the acoustic presence of any call type.

### Sediment trap data

Zooplankton samples were collected using two sediment traps on S1. Specifically, an automated PPS 3/3 Tecnicap sediment trap equipped with 12 cups (0,125 m^2^ collecting area) and a McLane sediment trap equipped with 13 cups (0.5 m² collecting area), respectively placed at 516 m and 1,002 m depth on the mooring line. The cup rotation intervals varied seasonally, ranging from 15 days to 1 month. Sediment trap cups were pre-filled with a buffered 5% (v/v) formaldehyde solution prepared in 0.45 μm filtered Arctic seawater to prevent organic matter (OM) degradation. After recovery, samples were stored in the dark at 4 °C and subsequently processed at the National Research Council—Institute of Polar Sciences (CNR-ISP) in Bologna, Italy. Mesozooplankton specimens, identified as swimmers (i.e., actively swimming zooplankton that enter the sediment trap funnel), were manually sorted under a Zeiss Discovery V8 stereomicroscope. Taxonomic identification was verified against the World Register of Marine Species (WoRMS) database to ensure accuracy. Fluxes of individuals were calculated as the number of individuals per collecting area per day.

### Environmental variables

To assess the environment and prey fields at a broader scale, daily concentration of zooplankton biomass (mmol/m^3^), concentration of phytoplankton biomass (mmol/m^3^), net primary production of biomass (mg/m^3^/day), and concentration of chlorophyll a (mg/m^3^) were downloaded from the Copernicus Global Ocean Biogeochemistry Analysis and Forecast (https://data.marine.copernicus.eu). The models provide a 6.25 km resolution. We extracted daily values for a 200 km x 200 km area around the hydrophone (nominally estimating that fin whale detections do not extend beyond 100 km in any direction). Daily forecasts were used to calculate monthly averages. Monthly sea ice area (km^2^) and extent (km^2^) for the Greenland Sea boundary (defined by the Arctic Sea Ice News and Analysis team using Meier et al. (2007)^112^ was downloaded from the National Snow and Ice Data Center’s (NSIDC) North Regional Sea Ice Monthly Data (https://nsidc.org/data/g02135)^113^. Because seasonal changes in daylight at the poles are more pronounced, we used getSunlightTimes in the “suncalc” package (v. 0.5.1)^114^ to summarize the number of sunlight minutes per month across the study period and assess diel trends in fin whale acoustic presence. Polar day was identified as times with 24 h of sun, polar night as times with 24 h of darkness, dawn as times before sunrise, day as times after sunrise but before sunset, dusk as times after sunset, and night s times after dusk.

### Statistical analysis

The “corrplot” package (v. 0.95)^115^ in R^58^ was used to assess correlation among abiotic and biotic environmental variables. The variables assessed included mean monthly concentrations of zooplankton, phytoplankton, net primary productivity, and chlorophyll a; sea ice area and extent; number of monthly sunlight minutes; number of taxa caught in the 516-m and 1,002-m sediment traps; combined number of taxa potentially relevant to fin whales (i.e., krill, copepods, and amphipods) caught in the 516-m sediment; and individual counts of krill, copepods, and amphipod taxa caught in the 516-m sediment trap. Only variables with a Pearson correlation coefficient (r) < 0.50 were retained.

Linear models were fitted using the package “stats” (v. 3.6.2) in R^58^ to determine whether there were differences in monthly percent acoustic presence by call type and abiotic and biotic environmental variables. The acoustic presence of individual call types was modeled as well as a combined call variable that included the acoustic presence of any call type. Model selection was done using *compare_performance()* in the package “performance” (v. 0.15.0.1)^116^, which computes Akaike’s Information Criteria (AIC), AIC Corrected (AICc), or Bayesian information criteria (BIC), R-squared (R^2^), Root Mean Squared Error (RMSE), and Sigma. Significance level was set at 0.05, but we report greater p-values (< 0.10) as a relationship in order to minimize the probability of Type II errors in studies with limited sample sizes^117^. Figures made in R^58^ with the package “ggplot2”^60^.

## Supplementary Information

Below is the link to the electronic supplementary material.

Supplementary Material 1

Supplementary Material 2

Supplementary Material 3

Supplementary Material 4

Supplementary Material 5

Supplementary Material 6

## Data Availability

Data and software supporting this research are restricted by government policies. Data and software are available in the CMRE servers, with access restricted only to governmental institutions of NATO member Nations and are not accessible to the public or research community. Should the need arise, the data set and software could be disclosed upon viable request via the establishment of a non-disclosure agreement. A formal request for the disclosure of the data can be addressed to NATO CMRE contact point via the official website https://www.cmre.nato.int/.
