# Supplementary material for: Year-round acoustic presence of fin whales southwest of Svalbard suggests mixed-use habitat for feeding and breeding
Source: Sci Rep. 2025 Nov 28;16:6845. doi: 10.1038/s41598-025-21785-x (PMC12916761; doi:10.1038/s41598-025-21785-x)
Supplement: Supplementary file 1 — Supplementary Material 1 [file 41598_2025_21785_MOESM1_ESM.docx]

**Year-round acoustic presence of fin whales around Svalbard suggests mixed-use habitat for feeding and breeding**

**Angela R Szesciorka^1^, Patrizia Giordano^2^, Manuel Bensi^3^, Alessandro Nicolai^2^, Aniello**

**Russo^1^, and Giacomo Giorli^1,*^**

^1^NATO Science and Technology Organization, Centre for Maritime Research and Experimentation, La Spezia, 19126, Italy

^2^National Research Council, Institute of Polar Sciences, Bologna, 40129, Italy

^3^National Institute of Oceanography and Applied Geophysics, Trieste, 34010, Italy

^*^Giacomo.Giorli@cmre.nato.int

**Supplementary Material**

**TABLES**

**Supplementary Table 1.** Model comparison from generalized linear models used to assess the relationship between 20 Hz, 130 Hz, downsweep, and any call type with uncorrelated variables (i.e., taxa in the 1,0002-m sediment trap and total number of krill, copepods, and amphipod in the 512-m sediment trap). Akaike’s Information Criteria (AIC), AIC Corrected (AICc), or Bayesian information criteria (BIC), R-squared (R^2^), Root Mean Squared Error (RMSE), and Sigma were used to choose the best-model (indicated by asterisk next to the run number for each call type).

| **Call Type** | **Run** | **variables** | **AIC (weights)** | **AICc (weights)** | **BIC (weights)** | **R^2^** | **RMSE** | **Sigma** |
| --- | --- | --- | --- | --- | --- | --- | --- | --- |
| 20 Hz | 1 | sed_trap_1000 + krill + copepods + amphipod | 104.7 (-0.203) | 121.5 (-0.006) | 107.6 (-0.172) | 0.811 | 11.523 | 15.087 |
| 20 Hz | 2 | sed_trap_1000 + krill + copepods + amphipod | 104.7 (-0.203) | 121.5 (-0.006) | 107.6 (-0.172) | 0.811 | 11.523 | 15.087 |
| 20 Hz | 3* | sed_trap_1000 + krill + copepods | 102.9 (-0.508) | 112.9 (-0.446) | 105.3 (-0.546) | 0.809 | 11.604 | 14.212 |
| 20 Hz | 4 | sed_trap_1000 + krill + amphipod | 115.5 (<.001) | 125.5 (<.001) | 117.9 (-0.001) | 0.454 | 19.6 | 24.005 |
| 20 Hz | 5 | sed_trap_1000 + copepods + amphipod | 111.6 (-0.006) | 121.6 (-0.006) | 114 (-0.007) | 0.604 | 16.69 | 20.441 |
| 20 Hz | 6 | krill + copepods + amphipod | 109 (-0.023) | 119 (-0.021) | 111.5 (-0.025) | 0.681 | 14.993 | 18.363 |
| 20 Hz | 7 | sed_trap_1000 + krill | 114.3 (-0.002) | 120 (-0.013) | 116.2 (-0.002) | 0.417 | 20.262 | 23.396 |
| 20 Hz | 8 | sed_trap_1000 + amphipod | 115.4 (<.001) | 121.1 (-0.007) | 117.3 (-0.001) | 0.36 | 21.226 | 24.51 |
| 20 Hz | 9 | sed_trap_1000 + copepods | 112.9 (-0.003) | 118.6 (-0.026) | 114.8 (-0.005) | 0.481 | 19.111 | 22.068 |
| 20 Hz | 10 | krill + amphipod | 116.3 (<.001) | 122 (-0.005) | 118.2 (<.001) | 0.311 | 22.015 | 25.421 |
| 20 Hz | 11 | krill + copepods | 108.1 (-0.038) | 113.8 (-0.285) | 110 (-0.052) | 0.652 | 15.653 | 18.075 |
| 20 Hz | 12 | amphipod + copepods | 111.8 (-0.006) | 117.5 (-0.044) | 113.7 (-0.008) | 0.525 | 18.289 | 21.118 |
| 20 Hz | 13 | sed_trap_1000 | 116.1 (<.001) | 119.1 (-0.02) | 117.6 (-0.001) | 0.195 | 23.797 | 26.068 |
| 20 Hz | 14 | krill | 116 (<.001) | 119 (-0.021) | 117.5 (-0.001) | 0.201 | 23.705 | 25.968 |
| 20 Hz | 15 | copepods | 113.8 (-0.002) | 116.8 (-0.063) | 115.3 (-0.004) | 0.337 | 21.606 | 23.668 |
| 20 Hz | 16 | amphipod | 115.2 (-0.001) | 118.2 (-0.031) | 116.6 (-0.002) | 0.256 | 22.889 | 25.074 |
| 130 Hz | 1 | sed_trap_1000 + krill + copepods + amphipod | 98.3 (-0.061) | 115.1 (<.001) | 101.2 (-0.041) | 0.662 | 8.814 | 11.541 |
| 130 Hz | 2 | sed_trap_1000 + krill + copepods + amphipod | 98.3 (-0.061) | 115.1 (<.001) | 101.2 (-0.041) | 0.662 | 8.814 | 11.541 |
| 130 Hz | 3 | sed_trap_1000 + krill + copepods | 96.8 (-0.126) | 106.8 (-0.014) | 99.3 (-0.107) | 0.646 | 9.018 | 11.045 |
| 130 Hz | 4 | sed_trap_1000 + krill + amphipod | 107.6 (<.001) | 117.6 (<.001) | 110 (<.001) | 0.136 | 14.103 | 17.273 |
| 130 Hz | 5 | sed_trap_1000 + copepods + amphipod | 97.8 (-0.077) | 107.8 (-0.009) | 100.2 (-0.065) | 0.616 | 9.396 | 11.508 |
| 130 Hz | 6 | krill + copepods + amphipod | 97.3 (-0.102) | 107.3 (-0.012) | 99.7 (-0.086) | 0.633 | 9.184 | 11.248 |
| 130 Hz | 7 | sed_trap_1000 + krill | 106.8 (<.001) | 112.5 (<.001) | 108.7 (<.001) | 0.046 | 14.819 | 17.111 |
| 130 Hz | 8 | sed_trap_1000 + amphipod | 105.6 (-0.002) | 111.3 (-0.002) | 107.5 (-0.002) | 0.135 | 14.107 | 16.29 |
| 130 Hz | 9 | sed_trap_1000 + copepods | 98.1 (-0.066) | 103.9 (-0.064) | 100.1 (-0.071) | 0.535 | 10.348 | 11.948 |
| 130 Hz | 10 | krill + amphipod | 105.9 (-0.001) | 111.6 (-0.001) | 107.8 (-0.001) | 0.113 | 14.287 | 16.497 |
| 130 Hz | 11* | krill + copepods | 95.5 (-0.25) | 101.2 (-0.245) | 97.4 (-0.27) | 0.627 | 9.259 | 10.692 |
| 130 Hz | 12 | amphipod + copepods | 97.2 (-0.107) | 102.9 (-0.105) | 99.1 (-0.115) | 0.571 | 9.939 | 11.477 |
| 130 Hz | 13 | sed_trap_1000 | 105.3 (-0.002) | 108.3 (-0.007) | 106.7 (-0.003) | 0.005 | 15.134 | 16.578 |
| 130 Hz | 14 | krill | 104.8 (-0.002) | 107.8 (-0.009) | 106.3 (-0.003) | 0.042 | 14.845 | 16.262 |
| 130 Hz | 15 | copepods | 96.7 (-0.136) | 99.7 (-0.517) | 98.1 (-0.187) | 0.513 | 10.588 | 11.599 |
| 130 Hz | 16 | amphipod | 103.9 (-0.004) | 106.9 (-0.014) | 105.4 (-0.005) | 0.11 | 14.309 | 15.675 |
| Downsweep | 1 | sed_trap_1000 + krill + copepods + amphipod | 93.4 (-0.01) | 110.2 (<.001) | 96.3 (-0.005) | 0.079 | 7.2 | 9.427 |
| Downsweep | 2 | sed_trap_1000 + krill + copepods + amphipod | 93.4 (-0.01) | 110.2 (<.001) | 96.3 (-0.005) | 0.079 | 7.2 | 9.427 |
| Downsweep | 3 | sed_trap_1000 + krill + copepods | 91.5 (-0.026) | 101.5 (-0.001) | 93.9 (-0.018) | 0.076 | 7.212 | 8.833 |
| Downsweep | 4 | sed_trap_1000 + krill + amphipod | 91.4 (-0.026) | 101.4 (-0.001) | 93.9 (-0.018) | 0.078 | 7.203 | 8.821 |
| Downsweep | 5 | sed_trap_1000 + copepods + amphipod | 92.1 (-0.019) | 102.1 (<.001) | 94.5 (-0.014) | 0.03 | 7.388 | 9.049 |
| Downsweep | 6 | krill + copepods + amphipod | 91.6 (-0.024) | 101.6 (-0.001) | 94 (-0.017) | 0.064 | 7.257 | 8.888 |
| Downsweep | 7 | sed_trap_1000 + krill | 89.5 (-0.07) | 95.2 (-0.028) | 91.4 (-0.062) | 0.076 | 7.213 | 8.329 |
| Downsweep | 8 | sed_trap_1000 + amphipod | 90.1 (-0.052) | 95.8 (-0.021) | 92 (-0.046) | 0.029 | 7.392 | 8.536 |
| Downsweep | 9 | sed_trap_1000 + copepods | 90.1 (-0.051) | 95.8 (-0.021) | 92.1 (-0.045) | 0.025 | 7.408 | 8.555 |
| Downsweep | 10 | krill + amphipod | 89.6 (-0.065) | 95.3 (-0.026) | 91.6 (-0.058) | 0.064 | 7.26 | 8.383 |
| Downsweep | 11 | krill + copepods | 89.7 (-0.061) | 95.5 (-0.025) | 91.7 (-0.055) | 0.055 | 7.295 | 8.424 |
| Downsweep | 12 | amphipod + copepods | 90.4 (-0.044) | 96.1 (-0.018) | 92.3 (-0.04) | 0.003 | 7.492 | 8.651 |
| Downsweep | 13 | sed_trap_1000 | 88.1 (-0.137) | 91.1 (-0.217) | 89.6 (-0.156) | 0.024 | 7.411 | 8.118 |
| Downsweep | 14* | krill | 87.7 (-0.166) | 90.7 (-0.262) | 89.2 (-0.189) | 0.055 | 7.295 | 7.991 |
| Downsweep | 15 | copepods | 88.4 (-0.12) | 91.4 (-0.189) | 89.9 (-0.136) | 0.002 | 7.496 | 8.212 |
| Downsweep | 16 | amphipod | 88.4 (-0.119) | 91.4 (-0.188) | 89.9 (-0.135) | 7.58E-04 | 7.5 | 8.216 |
| Any call | 1 | sed_trap_1000 + krill + copepods + amphipod | 103.8 (-0.199) | 120.6 (-0.005) | 106.7 (-0.167) | 0.823 | 11.069 | 14.492 |
| Any call | 2 | sed_trap_1000 + krill + copepods + amphipod | 103.8 (-0.199) | 120.6 (-0.005) | 106.7 (-0.167) | 0.823 | 11.069 | 14.492 |
| Any call | 3* | sed_trap_1000 + krill + copepods | 101.9 (-0.491) | 111.9 (-0.397) | 104.4 (-0.525) | 0.82 | 11.156 | 13.663 |
| Any call | 4 | sed_trap_1000 + krill + amphipod | 115.9 (<.001) | 125.9 (<.001) | 118.3 (<.001) | 0.427 | 19.916 | 24.392 |
| Any call | 5 | sed_trap_1000 + copepods + amphipod | 111.3 (-0.005) | 121.3 (-0.004) | 113.7 (-0.005) | 0.608 | 16.471 | 20.173 |
| Any call | 6 | krill + copepods + amphipod | 107.2 (-0.036) | 117.2 (-0.029) | 109.6 (-0.038) | 0.722 | 13.883 | 17.003 |
| Any call | 7 | sed_trap_1000 + krill | 114.7 (<.001) | 120.4 (-0.006) | 116.6 (-0.001) | 0.386 | 20.617 | 23.806 |
| Any call | 8 | sed_trap_1000 + amphipod | 115.7 (<.001) | 121.4 (-0.004) | 117.6 (<.001) | 0.333 | 21.494 | 24.819 |
| Any call | 9 | sed_trap_1000 + copepods | 112.7 (-0.002) | 118.4 (-0.016) | 114.6 (-0.003) | 0.48 | 18.974 | 21.909 |
| Any call | 10 | krill + amphipod | 116 (<.001) | 121.7 (-0.003) | 117.9 (<.001) | 0.315 | 21.787 | 25.157 |
| Any call | 11 | krill + copepods | 106.2 (-0.058) | 111.9 (-0.4) | 108.2 (-0.079) | 0.697 | 14.486 | 16.727 |
| Any call | 12 | amphipod + copepods | 110.9 (-0.006) | 116.6 (-0.039) | 112.8 (-0.008) | 0.552 | 17.607 | 20.33 |
| Any call | 13 | sed_trap_1000 | 116.4 (<.001) | 119.4 (-0.009) | 117.9 (<.001) | 0.16 | 24.118 | 26.42 |
| Any call | 14 | krill | 115.7 (<.001) | 118.7 (-0.013) | 117.2 (<.001) | 0.208 | 23.419 | 25.654 |
| Any call | 15 | copepods | 113 (-0.002) | 116 (-0.051) | 114.5 (-0.003) | 0.368 | 20.919 | 22.916 |
| Any call | 16 | amphipod | 115 (<.001) | 118 (-0.019) | 116.5 (-0.001) | 0.255 | 22.719 | 24.888 |

**FIGURES**

**
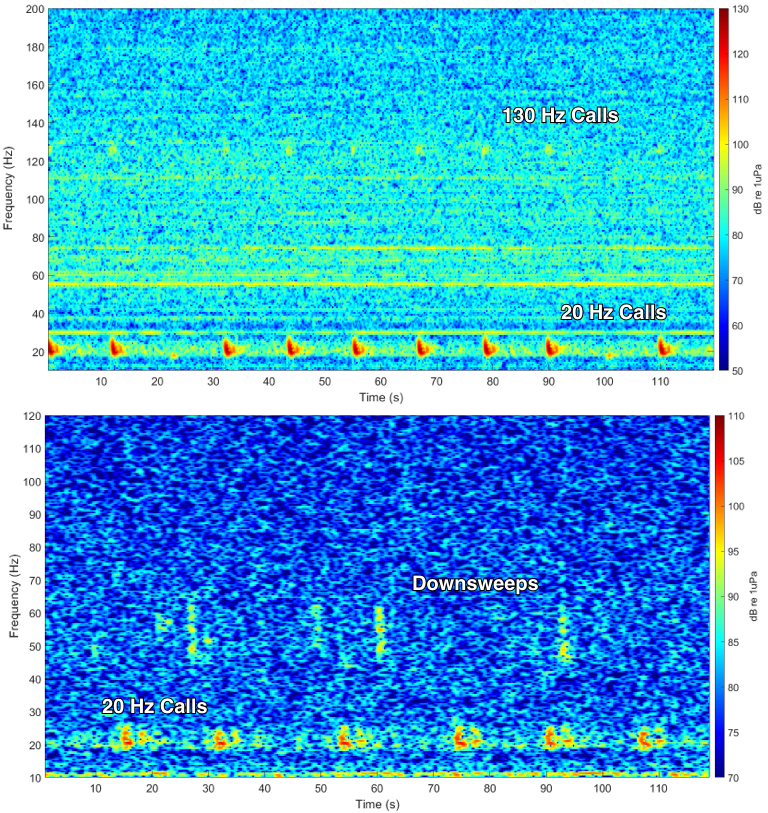
**

**Supplementary Figure 1.** Spectrograms of fin whale 20 Hz calls, 130 Hz calls, and downsweeps.


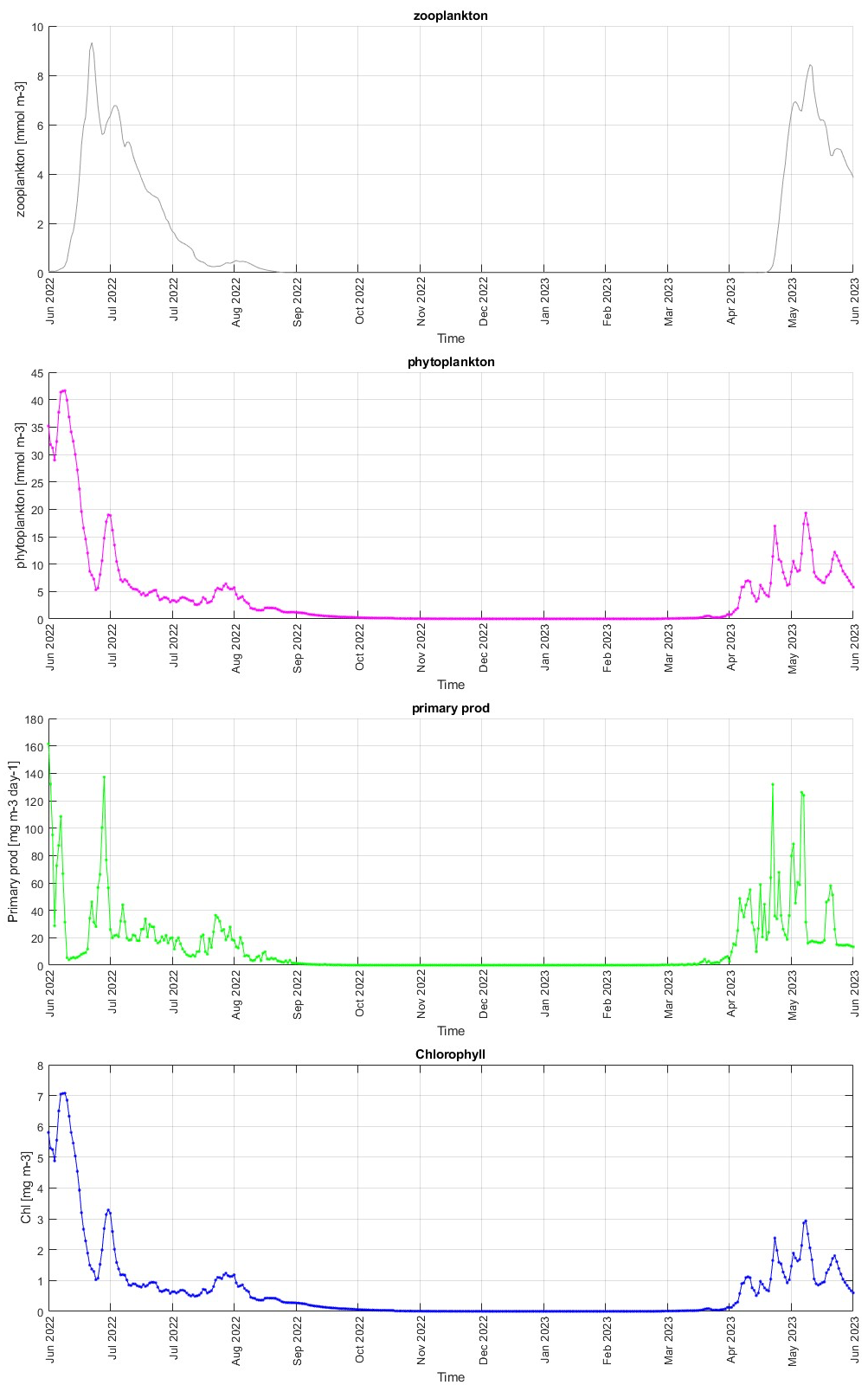


**Supplementary Figure 2.**  Daily timeseries of concentration of zooplankton biomass (mmol/m3), phytoplankton biomass (mmol/m3), net primary production of biomass (mg/m3/day), and chlorophyll a (mg/m3) in the 200 km x 200 km area around the S1 hydrophone.


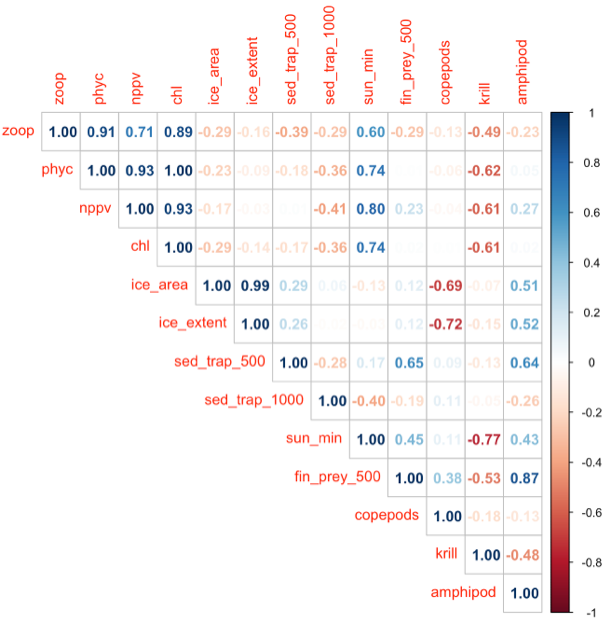


**Supplementary Figure 3.** Correlation matrix for the explanatory variables considered, including monthly mean concentration of zooplankton biomass (mmol/m^3^), mean concentration of phytoplankton biomass (mmol/m^3^), mean net primary production of biomass (mg/m^3^/day), and mean concentration of chlorophyll a (mg/m^3^), sea ice area (km^2^), sea ice extent (km^2^), number of taxa caught in the 516-m sediment trap, number of taxa caught in the 1,002-m sediment trap, number of monthly sunlight minutes, the number of taxa potentially relevant to fin whales (i.e., krill, copepods, and amphipods) caught in the 516-m sediment, number of krill taxa caught in the 516-m sediment trap, number of copepod taxa caught in the 516-m sediment trap, and number of amphipods caught in the 516-m sediment trap.


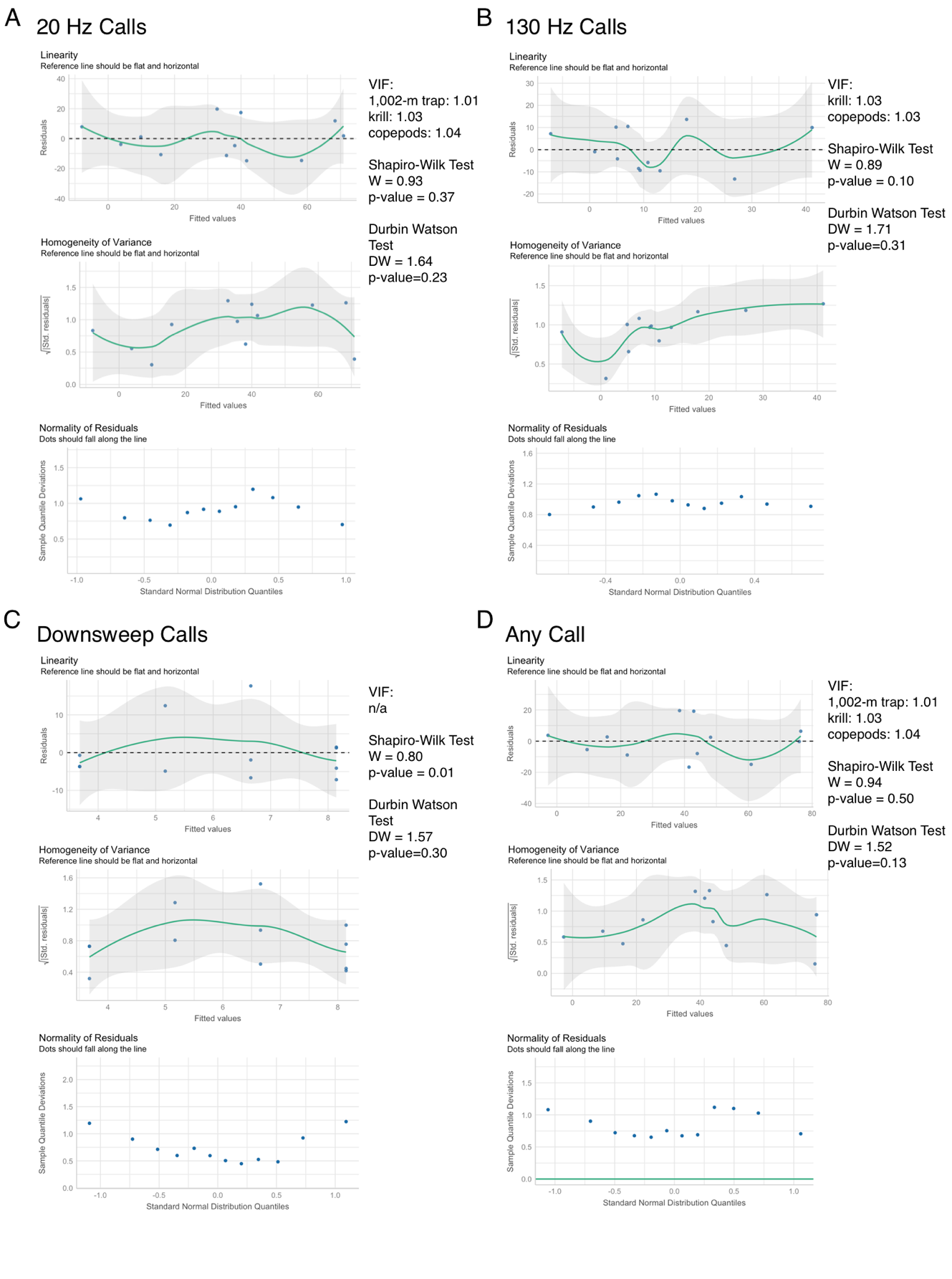


**Supplementary Figure 4.** Model diagnostics from best fitting models retained in model selection for 20 Hz calls, 130 Hz calls, downsweeps, and any call type.


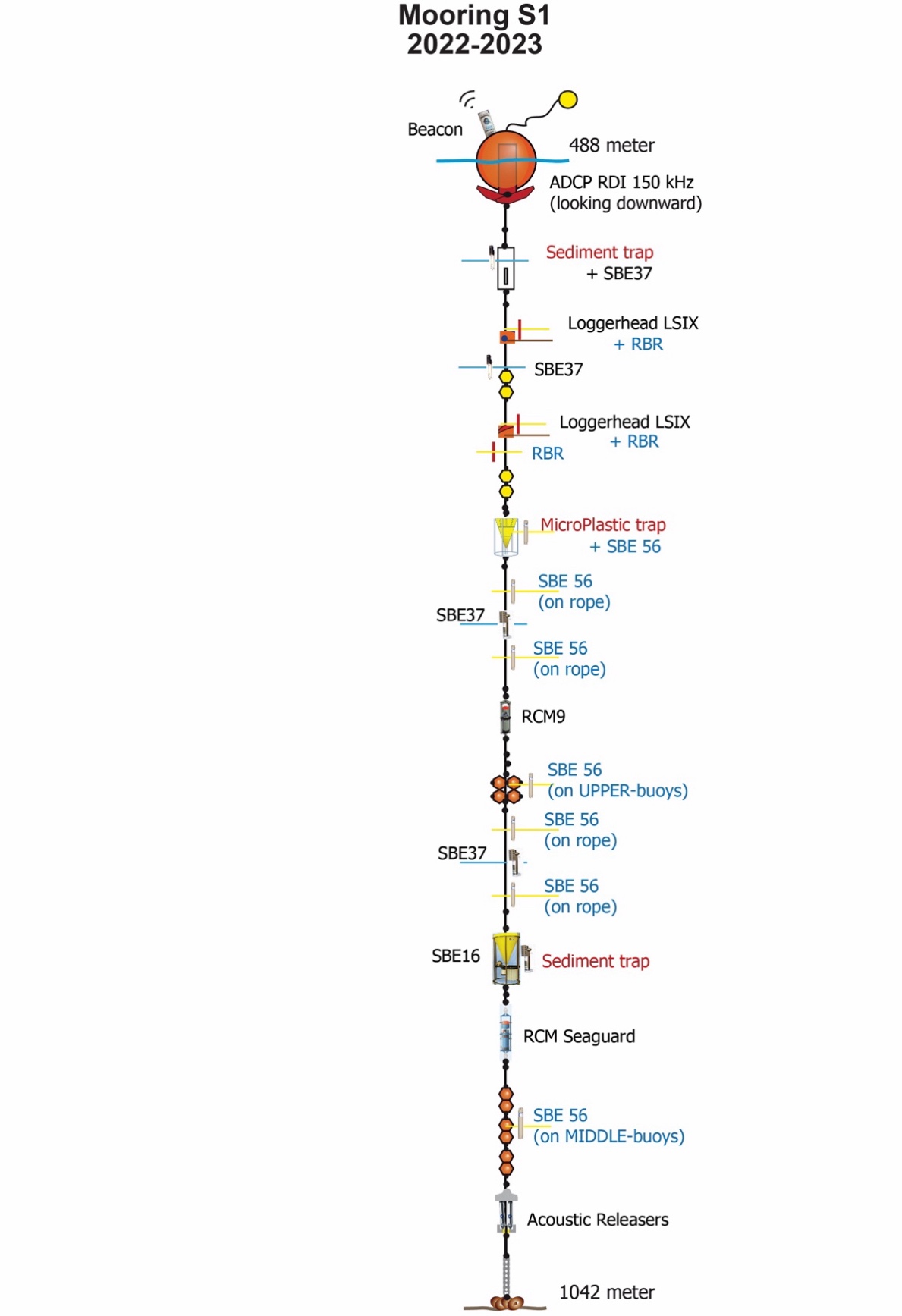


**Supplementary Figure 5**. Schematic of the S1 mooring deployed during NREP22 (2022/06–2023/06).
