# Supplementary figures and images for: Year-round acoustic presence of fin whales southwest of Svalbard suggests mixed-use habitat for feeding and breeding
Source: Sci Rep. 2025 Nov 28;16:6845. doi: 10.1038/s41598-025-21785-x (PMC12916761; doi:10.1038/s41598-025-21785-x)

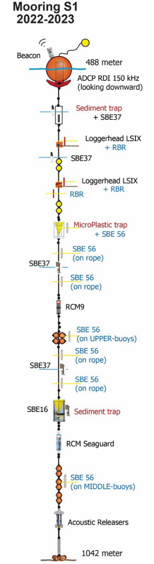

Supplement: Supplementary file 2 — Supplementary Material 2 [file 41598_2025_21785_MOESM2_ESM.tiff]

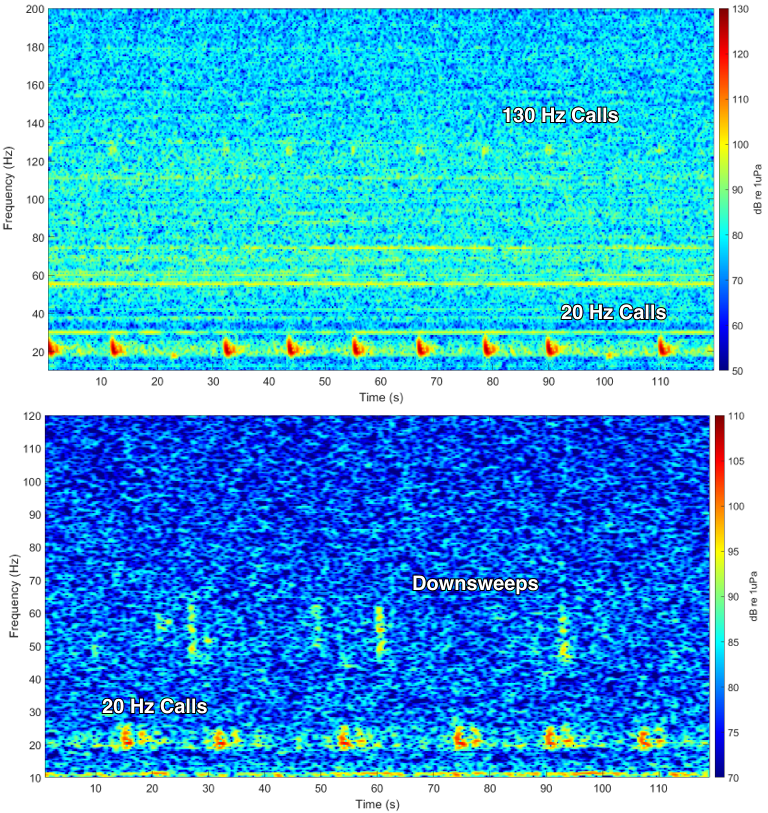

Supplement: Supplementary file 3 — Supplementary Material 3 [file 41598_2025_21785_MOESM3_ESM.tiff]

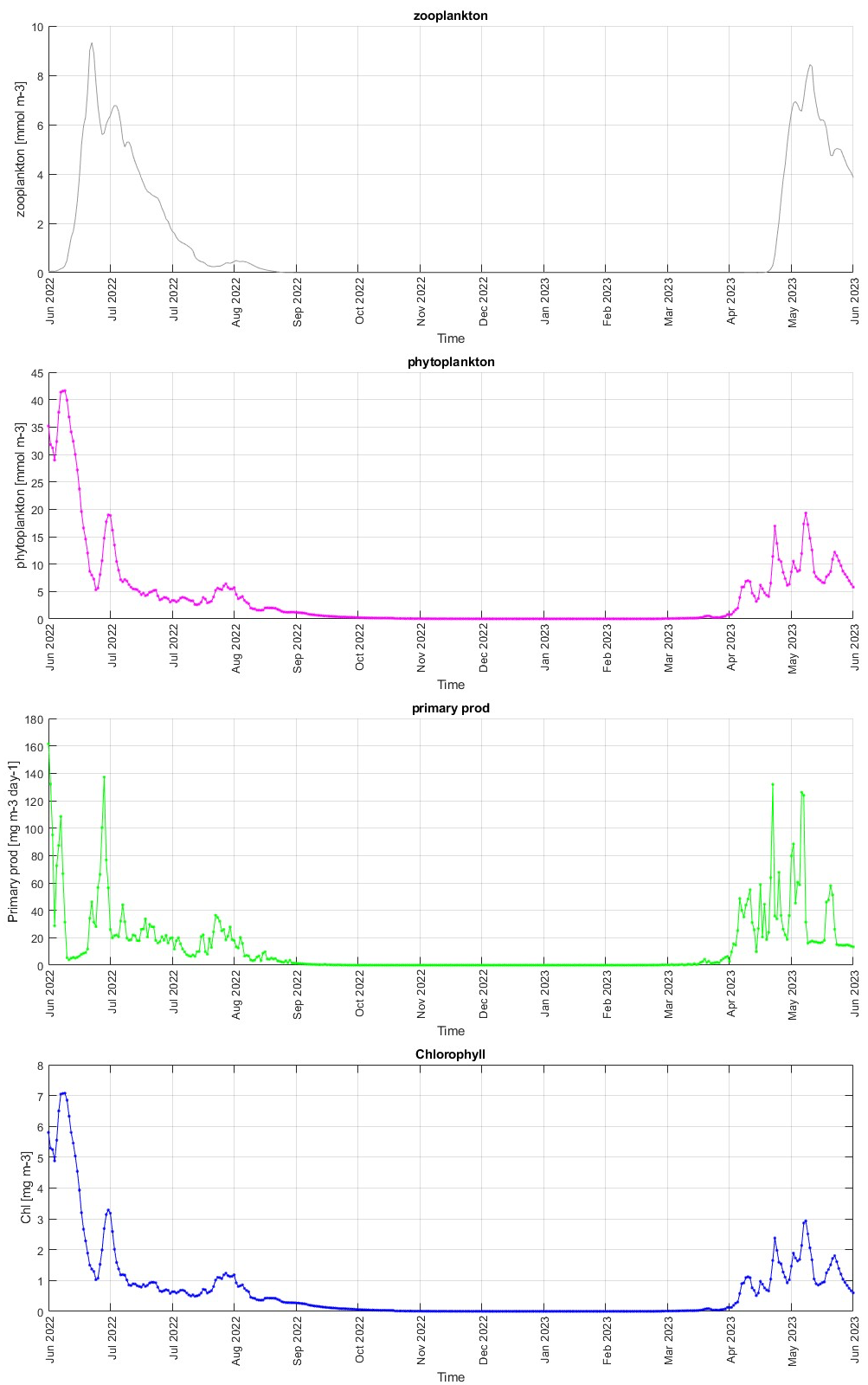

Supplement: Supplementary file 4 — Supplementary Material 4 [file 41598_2025_21785_MOESM4_ESM.tiff]

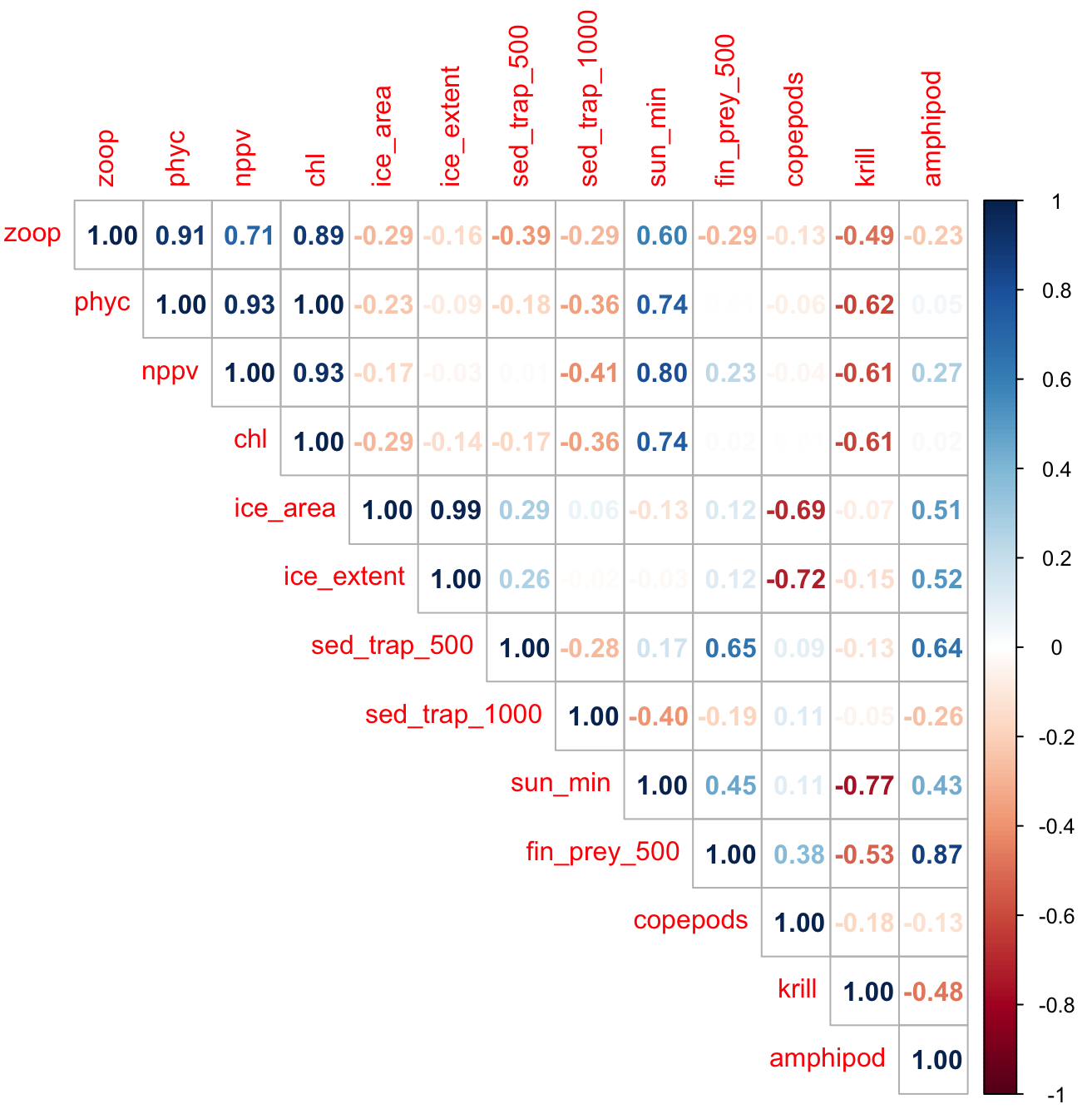

Supplement: Supplementary file 5 — Supplementary Material 5 [file 41598_2025_21785_MOESM5_ESM.tiff]

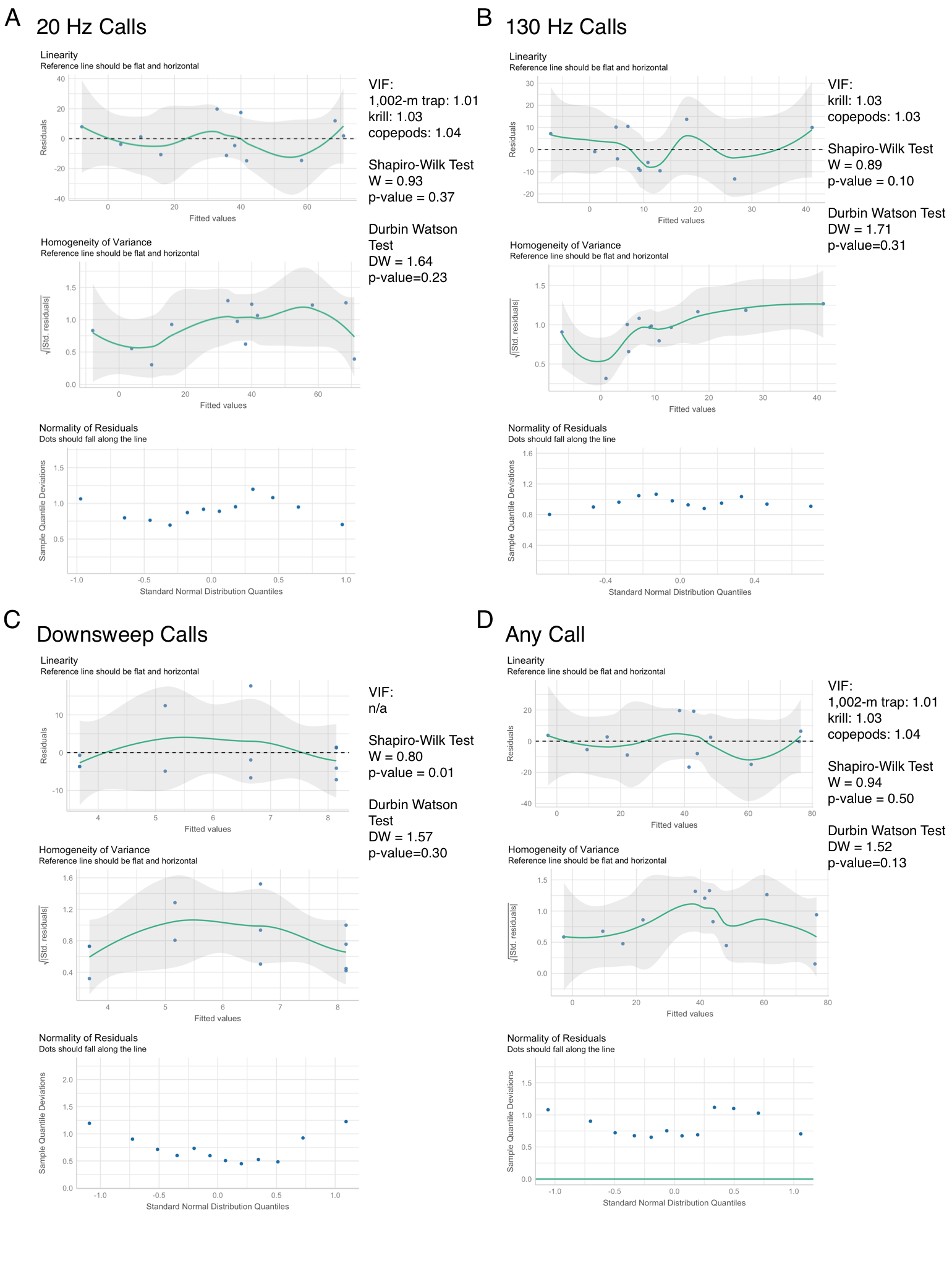

Supplement: Supplementary file 6 — Supplementary Material 6 [file 41598_2025_21785_MOESM6_ESM.tiff]
